# Infected pancreatic necrosis: outcomes and clinical predictors of mortality. A post hoc analysis of the MANCTRA-1 international study

**DOI:** 10.1007/s13304-023-01488-6

**Published:** 2023-03-11

**Authors:** Mauro Podda, Gianluca Pellino, Salomone Di Saverio, Federico Coccolini, Daniela Pacella, Stefano Piero Bernardo Cioffi, Francesco Virdis, Andrea Balla, Benedetto Ielpo, Francesco Pata, Gaetano Poillucci, Monica Ortenzi, Dimitrios Damaskos, Belinda De Simone, Massimo Sartelli, Ari Leppaniemi, Kumar Jayant, Fausto Catena, Antonio Giuliani, Marcello Di Martino, Adolfo Pisanu, the MANCTRA-1 Collaborative Group, Chiara Gerardi, Stavros Gourgiotis, Cristiana Riboni, Alessio Giordano, Luca Ferrario, Vanni Agnoletti, Yoram Kruger, Damian Mole, Ferdinando Agresta, Mikel Prieto Calvo, Michael Wilson, Fiammetta Soggiu, Alaa Hamdan, Carlos Augusto Gomes, Gustavo Fraga, Argyrios Ioannidis, Zaza Demetrashvili, Saaz Sahani, Lovenish Bains, Almu’atasim Khamees, Hazim Ababneh, Osama Aljaiuossi, Samuel Pimentel, Ikhwan Sani Mohamad, Ahmad Ramzi Yusoff, Narcis Octavian Zarnescu, Valentin Calu, Andrey Litvin, Dusan Lesko, Ahmed Elmehrath, Mohamedraed Elshami, Martin de Santibañes, Justin Gundara, Kamel Alawadhi, Rashid Lui, Alexander Julianov, Sergio Ralon, Ibrahim-Umar Garzali, Gustavo M Machain, Ibabe Villalabeitia, Darwin Artidoro Quispe-Cruz, Abigail Cheska C. Orantia, Maciej Walędziak, Tiago Correia de Sá, Syed Muhammad Ali, Bojan Kovacevic, Colin Noel, Haidar M. Abdalah, Ali Kchaou, Arda Isik, Luca Ansaloni, Walter Biffl, Mario Guerrieri, Alberto Sartori, Manuel Abradelo, Giuseppe Nigri, Nicola Di Lorenzo, Andrea Mingoli, Massimo Chiarugi, Juliana Di Menno Stavron, Oscar Mazza, José Ignacio Valenzuela, Diana Alejandra Pantoja Pachajoa, Fernando Andrés Alvarez, Julian Ezequiel Liaño, Joan Tefay, Abdulrahman Alshaikh, Layla Hasan, Felipe Couto Gomes, Thiago R. A. Calderan, Elcio S. Hirano, Dragomir Dardanov, Alexander Julianov, Azize Saroglu, Boyko Atanasov, Nikolay Belev, Nikola Kovachev, Shannon Melissa Chan, Hon-Ting Lok, Diego Salcedo, Diana Robayo, María Alejandra Triviño, Jan Manak, Saaz Sahani, Jorann de Araujo, Ananya Sethi, Ahmed Awad, Merihan Elbadawy, Ahmed Farid, Asmaa Hanafy, Ahmed Nafea, Ghozy Sherief, Abbas Salah Alzhraa, Wafaa Abdelsalam, Sameh Emile, Ahmed Elfallal, Hossam Elfeki, Hosam Elghadban, Ashraf Shoma, Mohamed Shetiwy, Mohamed Elbahnasawy, Salem Mohamed, Emad Fawzi Hamed, Usama Ahmed Khalil, Elie Chouillard, Andrew Gumbs, Andrea Police, Andrea Mabilia, Kakhi Khutsishvili, Anano Tvaladze, Orestis Ioannidis, Elissavet Anestiadou, Lydia Loutzidou, Konstantinis Konstantinidis, Sofia Konstantinidou, Dimitrios Manatakis, Vasileios Acheimastos, Nikolaos Tasis, Nikolaos Michalopoulos, Panagiotis Kokoropoulos, Maria Papadoliopoulou, Maria Sotiropoulou, Stylianos Kapiris, Panagiotis Metaxas, Ioannis Tsouknidas, Despoina Kefili, George Petrakis, Eirini Synekidou, Konstantinos Dakis, Eirini Alexandridou, Aristeidis Papadopoulos, Christos Chouliaras, Odysseas Mouzakis, Francesk Mulita, Ioannis Maroulis, Michail Vailas, Tania Triantafyllou, Dimitrios Theodorou, Eftychios Lostoridis, Eleni-Aikaterini Nagorni, Paraskevi Tourountzi, Efstratia Baili, Alexandros Charalabopoulos, Theodore Liakakos, Dimitrios Schizas, Alexandros Kozadinos, Athanasios Syllaios, Nikolaos Machairas, Stylianos Kykalos, Paraskevas Stamopoulos, Spiros Delis, Christos Farazi-Chongouki, Evangelos Kalaitzakis, Miltiadis Giannarakis, Konstantinos Lasithiotakis, Giorgia Petra, Evangelos Kalaitzakis, Amit Gupta, Noushif Medappil, Vijayanand Muthukrishnan, Jubin Kamar, Pawan Lal, Rajendra Agarwal, Matteo Magnoli, Paolo Aonzo, Alberto Serventi, Pierpaolo Di Lascio, Margherita Pinto, Carlo Bergamini, Andrea Bottari, Laura Fortuna, Jacopo Martellucci, Atea Cicako, Claudio Miglietta, Mario Morino, Daniele Delogu, Andrea Picchetto, Marco Assenza, Giancarlo D’Ambrosio, Giulio Argenio, Mariano Fortunato Armellino, Giovanna Ioia, Savino Occhionorelli, Dario Andreotti, Lacavalla Domenico, Davide Luppi, Massimiliano Casadei, Luca Di Donato, Farshad Manoochehri, Tiziana Rita Lucia Marchese, William Sergi, Roberto Manca, Raimondo Murgia, Enrico Piras, Lorenzo Conti, Simone Gianazza, Andrea Rizzi, Edoardo Segalini, Marco Monti, Elena Iiritano, Nicolò Maria Mariani, Enrico De Nicola, Giovanna Scifo, Giusto Pignata, Jacopo Andreuccetti, Francesco Fleres, Guglielmo Clarizia, Alessandro Spolini, Alan Biloslavo, Paola Germani, Manuela Mastronardi, Selene Bogoni, Silvia Palmisano, Nicolo’ De Manzini, Marco Vito Marino, Gennaro Martines, Giuseppe Trigiante, Elpiniki Lagouvardou, Gabriele Anania, Cristina Bombardini, Dario Oppici, Tiziana Pilia, Valentina Murzi, Emanuela Gessa, Umberto Bracale, Maria Michela Di Nuzzo, Roberto Peltrini, Francesco Salvetti, Jacopo Viganò, Gabriele Sganga, Valentina Bianchi, Pietro Fransvea, Tommaso Fontana, Giuliano Sarro, Vincenza Paola Dinuzzi, Luca Scaravilli, Mario Virgilio Papa, Elio Jovine, Giulia Ciabatti, Laura Mastrangelo, Matteo Rottoli, Claudio Ricci, Iris Shari Russo, Alberto Aiolfi, Davide Bona, Francesca Lombardo, Pasquale Cianci, Mariagrazia Sederino, Roberto Bini, Osvaldo Chiara, Stefano Cioffi, Stefano Cantafio, Guido Coretti, Edelweiss Licitra, Grazia Savino, Sergio Grimaldi, Raffaele Porfidia, Elisabetta Moggia, Mauro Garino, Chiara Marafante, Antonio Pesce, Nicolò Fabbri, Carlo Vittorio Feo, Ester Marra, Marina Troian, Davide Drigo, Carlo Nagliati, Andrea Muratore, Riccardo Danna, Alessandra Murgese, Michele Crespi, Claudio Guerci, Alice Frontali, Luca Ferrari, Claudio Guerci, Francesco Favi, Erika Picariello, Alessia Rampini, Fabrizio D’Acapito, Giorgio Ercolani, Leonardo Solaini, Francesco Palmieri, Matteo Calì, Francesco Ferrara, Irnerio Angelo Muttillo, Edoardo Maria Muttillo, Biagio Picardi, Raffaele Galleano, Ali Badran, Omar Ghazouani, Maurizio Cervellera, Gaetano Campanella, Gennaro Papa, Annamaria Di Bella, Gennaro Perrone, Gabriele Luciano Petracca, Concetta Prioriello, Mario Giuffrida, Federico Cozzani, Matteo Rossini, Marco Inama, Giovanni Butturini, Gianluigi Moretto, Luca Morelli, Giulio Candio, Simone Guadagni, Enrico Cicuttin, Camilla Cremonini, Dario Tartaglia, Valerio Genovese, Nicola Cillara, Alessandro Cannavera, Antonello Deserra, Arcangelo Picciariello, Vincenzo Papagni, Leonardo Vincenti, Giulia Bagaglini, Giuseppe Sica, Pierfrancesco Lapolla, Gioia Brachini, Dario Bono, Antonella Nicotera, Marcello Zago, Fabrizio Sammartano, Laura Benuzzi, Marco Stella, Stefano Rossi, Alessandra Cerioli, Caterina Puccioni, Stefano Olmi, Carolina Rubicondo, Matteo Uccelli, Anna Guida, Pasquale Lepiane, Diego Sasia, Giorgio Giraudo, Sara Salomone, Elena Belloni, Alessandra Cossa, Francesco Lancellotti, Roberto Caronna, Piero Chirletti, Paolina Saullo, Raffaele Troiano, Felice Mucilli, Mirko Barone, Massimo Ippoliti, Michele Grande, Bruno Sensi, Leandro Siragusa, Andrea Santini, Isidoro Di Carlo, Massimiliano Veroux, Rossella Gioco, Gastone Veroux, Giuseppe Currò, Michele Ammendola, Iman Komaei, Giuseppe Navarra, Valeria Tonini, Lodovico Sartarelli, Marco Ceresoli, Stefano Perrone, Linda Roccamatisi, Paolo Millo, Riccardo Brachet Contul, Elisa Ponte, Matteo Zuin, Giuseppe Portale, Alice Sabrina Tonello, Geri Fratini, Matteo Bianchini, Bruno Perotti, Emanuele Doria, Elia Giuseppe Lunghi, Diego Visconti, Khayry Al-Shami, Sajeda Awadi, Mohammad Musallam Khalil Buwaitel, Mo’taz Fawzat Naief Naffa’, Ahmad Samhouri, Hatem Sawalha, Mohd Firdaus Che Ani, Ida Nadiah Ahmed Fathil, Jih Huei, Ikhwan Sani Mohamad, Jose-Luis Beristain-Hernandez, Alejandro Garcia-Meza, Rafael Sepulveda-Rdriguez, Edgard Efren Lozada Hernández, Camilo Levi Acuña Pinzón, Jefferson Nieves Condoy, Francisco C. Becerra García, Mohammad Sadik, Bushra Kadir, Jalpa Devi, Nandlal Seerani, Zainab, Mohammad Sohail-Asghar, Ameer Afzal, Ali Akbar, Helmut Segovia Lohse, Herald Segovia Lohse, Zamiara Solange Leon Cabrera, Gaby Susana Yamamoto Seto, José Ríos Chiuyari, Jorge Ordemar, Martha Rodríguez, Abigail Cheska C. Orantia-Carlos, Margie Antionette Quitoy, Andrzej Kwiatkowski, Maciej Mawlichanów, Mónica Rocha, Carlos Soares, Alexandru Rares Stoian, Andreea Diana Draghici, Valentin Titus Grigorean, Raluca Bievel Radulescu, Narcis Octavian Zarnescu, Radu Virgil Costea, Eugenia Claudia Zarnescu, Mikhail Kurtenkov, George Gendrikson, Volovich Alla-Angelina, Tsurbanova Arina, Ayrat Kaldarov, Ayrat Kaldarov, Mahir Gachabayov, Abakar Abdullaev, Milica Milentijevic, Milovan Karamarkovic, Arpád Panyko, Jozef Radonak, Marek Soltes, Laura Álvarez Morán, Haydée Calvo García, Pilar Suárez Vega, Sergio Estevez, Fabio Ausania, Jordi Farguell, Carolina González-Abós, Santiago Sánchez-Cabús, Belén Martín, Víctor Molina, Luis Oms, Lucas Ilzarbe, Eva Pont Feijóo, Elena Sofia Perra, Noel Rojas-Bonet, Rafael Penalba-Palmí, Susana Pérez-Bru, Jaume Tur-Martínez, Andrea Álvarez-Torrado, Marta Domingo-Gonzalez, Javier Tejedor-Tejada, Yaiza García del Alamo, Fernando Mendoza-Moreno, Francisca García-Moreno-Nisa, Belén Matías-García, Manuel Durán, Rafael Calleja-Lozano, José Manuel Perez de Villar, Luis Sánchez-Guillén, Iban Caravaca, Daniel Triguero-Cánovas, Antonio Carlos Maya Aparicio, Blas Durán Meléndez, Andrea Masiá Palacios, Aitor Landaluce-Olavarria, Mario De Francisco, Begoña Estraviz-Mateos, Felipe Alconchel, Tatiana Nicolás-López, Pablo Ramírez, Virginia Duran Muñoz-Cruzado, Felipe Parej Ciuró, Eduardo Perea del Pozo, Sergio Olivares Pizarro, Vicente Herrera Cabrera, Jose Muros Bayo, Hytham K. S. Hamid, Raffaello Roesel, Alessandra Cristaudi, Kinan Abbas, Iyad Ali, Ahmed Tlili, Hüseyin Bayhan, Mehmet Akif Türkoğlu, Mustafa Yener Uzunoglu, Ibrahim Fethi Azamat, Nail Omarov, Derya Salim Uymaz, Fatih Altintoprak, Emrah Akin, Necattin First, Koray Das, Nazmi Ozer, Ahmet Seker, Yasin Kara, Mehmet Abdussamet Bozkurt, Ali Kocataş, Semra Demirli Atici, Murat Akalin, Bulent Calik, Elif Colak, Yuksel Altinel, Serhat Meric, Yunus Emre Aktimur, Victoria Hudson, Jean-Luc Duval, Mansoor Khan, Ahmed Saad, Mandeep Kaur, Alison Bradley, Katherine Fox, Ivan Tomasi, Daniel Beasley, Alekhya Kotta Prasanti, Pinky Kotecha, Husam Ebied, Michaela Paul, Hemant Sheth, Ioannis Gerogiannis, Mohannad Gaber, Zara Sheikh, Shatadru Seth, Maria Kunitsyna, Cosimo Alex Leo, Vittoria Bellato, Noman Zafar, Amr Elserafy, Giles Bond-smith, Giovanni Tebala, Pawan Mathur, Izza Abid, Nnaemeka Chidumije, Pardip Sandhar, Syed Osama Zohaib Ullah, Tamara Lezama, Muhammad Hassan Anwaar, Conor Magee, Salma Ahmed, Brooke Davies, Jeyakumar Apollos, Kieran McCormack, Hasham Choudhary, Triantafyllos Doulias, Tamsin Morrison, Anna Palepa, Fernando Bonilla Cal, Lianet Sánchez, Fabiana Domínguez, Ibrahim Al-Raimi, Haneen Alshargabi, Abdullah Meead, Serge Chooklin, Serhii Chuklin, Andriy Bilyak

**Affiliations:** 1 Emergency Surgery Unit, Department of Surgical Science, Policlinico Universitario “D. Casula”, Azienda Ospedaliero-Universitaria di Cagliari, University of Cagliari, SS 554, Km 4,500, Monserrato, 09042 Cagliari, Italy; 2 Department of Advanced Medical and Surgical Sciences, Università degli Studi della Campania “Luigi Vanvitelli”, Naples, Italy; 3 Colorectal Surgery Unit, Vall d’Hebron University Hospital, Barcelona, Spain; 4 Department of Surgery, “Madonna del Soccorso” Hospital, San Benedetto del Tronto, Italy; 5 General, Emergency and Trauma Surgery Unit, Pisa University Hospital, Pisa, Italy; 6 Department of Public Health, University of Naples Federico II, Naples, Italy; 7 Trauma and Acute Care Surgery Unit, “Niguarda Ca Granda” Hospital, Milan, Italy; 8 General and Minimally-Invasive Surgery Unit, “San Paolo” Hospital, Civitavecchia, Rome, Italy; 9 HPB Surgery Unit, Hospital del Mar, Barcelona, Spain; 10 General Surgery Unit, “Nicola Giannettasio” Hospital, Corigliano-Rossano, Italy; 11 Department of General Surgery, Policlinico Umberto I, La Sapienza University of Rome, Rome, Italy; 12 Department of General and Emergency Surgery, Marche Polytechnic University, Ancona, Italy; 13 Department of Upper G.I. Surgery, Royal Infirmary of Edinburgh, Edinburgh, Scotland, UK; 14 Department of Emergency and Metabolic Minimally Invasive Surgery, Centre Hospitalier Intercommunal de Poissy/Saint Germain en Laye, Poissy Cedex, France; 15 Department of Surgery, Macerata Civil Hospital, Macerata, Italy; 16 Department of Abdominal Surgery, Abdominal Center, University of Helsinki and Helsinki University Central Hospital, Helsinki, Finland; 17 Department of Surgery & Cancer, Imperial College London, Du Cane Road, London, UK; 18 Department of Emergency and Trauma Surgery, “Bufalini” Hospital, Cesena, Italy; 19 General and Emergency Surgery Unit, San Carlo Hospital, Potenza, Italy; 20 Division of Hepatobiliary and Liver Transplantation Surgery, “A.O.R.N. Cardarelli”, Naples, Italy

**Keywords:** Acute pancreatitis, Infected pancreatic necrosis, International study, Organ failure, Mortality

## Abstract

The identification of high-risk patients in the early stages of infected pancreatic necrosis (IPN) is critical, because it could help the clinicians to adopt more effective management strategies. We conducted a post hoc analysis of the MANCTRA-1 international study to assess the association between clinical risk factors and mortality among adult patients with IPN. Univariable and multivariable logistic regression models were used to identify prognostic factors of mortality. We identified 247 consecutive patients with IPN hospitalised between January 2019 and December 2020. History of uncontrolled arterial hypertension (*p* = 0.032; 95% CI 1.135–15.882; aOR 4.245), qSOFA (*p* = 0.005; 95% CI 1.359–5.879; aOR 2.828), renal failure (*p* = 0.022; 95% CI 1.138–5.442; aOR 2.489), and haemodynamic failure (*p* = 0.018; 95% CI 1.184–5.978; aOR 2.661), were identified as independent predictors of mortality in IPN patients. Cholangitis (*p* = 0.003; 95% CI 1.598–9.930; aOR 3.983), abdominal compartment syndrome (*p* = 0.032; 95% CI 1.090–6.967; aOR 2.735), and gastrointestinal/intra-abdominal bleeding (*p* = 0.009; 95% CI 1.286–5.712; aOR 2.710) were independently associated with the risk of mortality. Upfront open surgical necrosectomy was strongly associated with the risk of mortality (*p* < 0.001; 95% CI 1.912–7.442; aOR 3.772), whereas endoscopic drainage of pancreatic necrosis (*p* = 0.018; 95% CI 0.138–0.834; aOR 0.339) and enteral nutrition (p = 0.003; 95% CI 0.143–0.716; aOR 0.320) were found as protective factors. Organ failure, acute cholangitis, and upfront open surgical necrosectomy were the most significant predictors of mortality. Our study confirmed that, even in a subgroup of particularly ill patients such as those with IPN, upfront open surgery should be avoided as much as possible. Study protocol registered in ClinicalTrials.Gov (I.D. Number NCT04747990).

**Graphical abstract:**

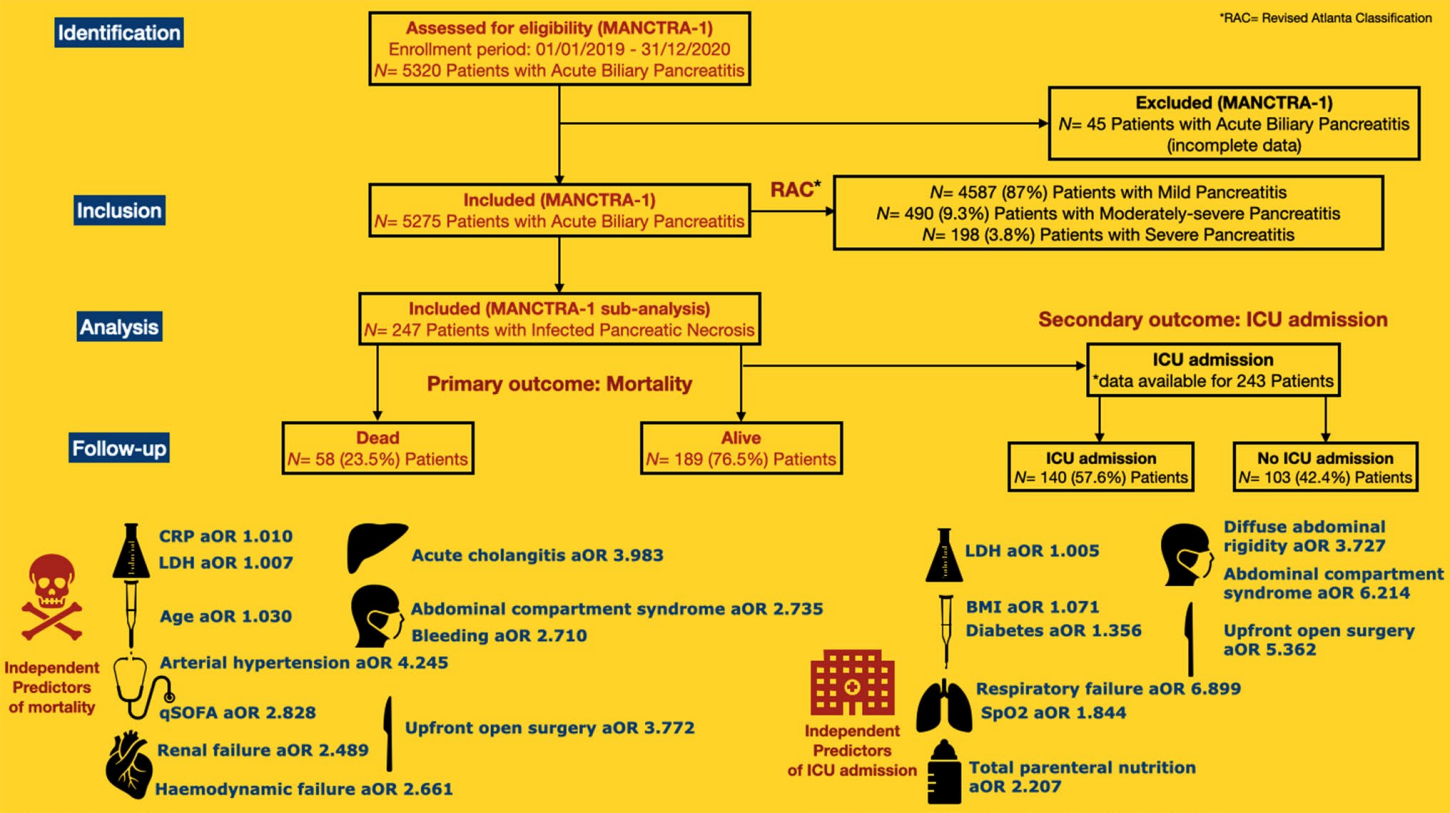

**Supplementary Information:**

The online version contains supplementary material available at 10.1007/s13304-023-01488-6.

## Introduction

With an incidence of about 34 cases per 100,000 people, acute pancreatitis (AP) is the most frequent non-malignant gastroenterological disorder leading to hospitalisation worldwide [1–3]. Although 80% of AP patients have a mild self-limited clinical course, the other 20% will develop severe AP, characterised by pancreatic necrosis and organ failure, with a 35–50% mortality rate [4].

The early clinical course of severe AP is characterised by a dysregulated systemic inflammatory response syndrome, organ dysfunction, and acute fluid or necrotic collections. After recovery from the acute phase, 20% of patients present necrosis involving the pancreatic parenchyma, the surrounding fatty tissue, or both. While most necrotic collections remain sterile, about 30% of these patients will develop a superimposed necrosis infection, which is usually diagnosed by the presence of gas in the collections, positive culture of the pancreatic necrosis aspirate, and persistent sepsis or ongoing clinical deterioration. Prognostic factors associated with the development of infected pancreatic necrosis (IPN) in patients with acute necrotising or severe AP include older age, gallstone aetiology, greater than 50% necrosis of the pancreas, delayed enteral nutrition, multiple or persistent organ failure, and invasive mechanical ventilation [5]. Established scores such as the APACHE II and Ranson’s have been proposed to grade disease severity and predict mortality. Similarly, several laboratory parameters, such as inflammatory markers, kidney function tests, and haematocrit have been trialled to accurately predict severe AP, development of necrosis and mortality [6]. Patients with IPN have been found to have higher APACHE II scores and higher values of lipase, C-reactive protein, and procalcitonin compared to patients with sterile necrosis [7].

With a mortality rate of up to 35%, IPN carries the clinical challenge of working with a multidisciplinary approach, determining proper timing for interventions, and identifying appropriate treatment strategies based on individual patient anatomy, pathophysiology, and local expertise [8–16]. Over the last decade, standard treatments of IPN have shifted from open surgical necrosectomy towards the so-called "step-up" endoscopic and percutaneous/minimally invasive approaches [17–22].

In the study by Wu et al*.* [23] aiming to investigate the risk factors for mortality among the population of patients with IPN, sequential organ failure assessment (SOFA) score > 2 and procalcitonin > 6 ng/L were independent predictors of mortality. Prognostic factors associated with the development of IPN in patients with acute necrotising or severe AP have been defined; on the other hand, although established scores such as the APACHE II and Ranson’s have been used to predict mortality, the predictors of an increased mortality rate in those patients who develop IPN have not been described yet.

### Study aim

Considering the high mortality rates associated with IPN, the identification of high-risk patients in the early stage of the disease (within 48–72 from hospital admission) is critical as it can help clinicians guide aggressive interventions and institute more effective management strategies to improve the prognosis. Thus, we conducted a post hoc analysis of the coMpliAnce with evideNce-based cliniCal guidelines in the managemenT of acute biliaRy pancreAtitis (MANCTRA-1) international study [24] to assess the association between clinical risk factors present early from hospital admission (within 72 h) and the subsequent development of fatal complications among adult patients with IPN, to implement potential mitigation strategies and improve survival outcomes.

## Methods

### Study design

The present study is a post hoc analysis of the MANCTRA-1 study, conducted in 150 centres in Europe, Asia, Africa, South America and Oceania [24, 25]. Ethical approval of the MANCTRA-1 study and subsequent post hoc analyses was granted by the Institutional Review Board of the University of Cagliari (Italy) (PROT. P.G./ 2021/5410–31/03/2021) and local boards of the participating centres. This study was conducted under the principles of the Declaration of Helsinki and was developed and presented according to Strengthening the Reporting of Observational Studies in Epidemiology (STROBE, ClinicalTrials. Gov NCT04747990) [26]. A retrospective analysis was performed on all consecutive patients hospitalised between January 2019 and December 2020 with a diagnosis of IPN associated with biliary pancreatitis. The exclusion criteria were the following: age younger than 16 years, patients with AP having an aetiology other than gallstones, history of chronic pancreatitis, pregnancy, or breastfeeding women.

### Definitions

Necrotizing AP was defined as a lack of pancreatic parenchyma enhancement and/or findings of extra-pancreatic necrosis on contrast-enhanced computed tomography (CT) scan [14]. IPN was defined as contrast-enhanced CT scan evidence of gas collections in the pancreatic and/or extra-pancreatic tissues with evidence of sepsis identified with the increase of C-reactive protein (CRP) and procalcitonin, associated with fever and increased leukocytosis, abdominal pain, and deterioration of the clinical parameters and/or a positive culture of pancreatic necrosis obtained by fine-needle aspiration (FNA), percutaneous or endoscopic drainage or necrosectomy. Comorbidity was calculated on admission using the Charlson Comorbidity Index (CCI). The patients were classified as having severe AP based on persistent organ failure for more than 48 h, according to the revised Atlanta classification (RAC). Organ failure was defined as follows: respiratory failure (partial pressure of arterial oxygen-paO_2_- < 60 mm), acute renal failure (serum creatinine > 2.0 mg/dL), haemodynamic failure (systolic blood pressure < 90 mmHg) any time during the first 72 h of hospital admission [14]. Obesity was defined according to the Centers for Disease Control (CDC) as patients with body mass index (BMI) > 30 kg/m^2^. Abdominal compartment syndrome (ACS) was reported based on the World Society of the Abdominal Compartment Syndrome definition of sustained intra-abdominal pressure (IAP) > 20 mmHg associated with new organ dysfunction [27]. In-hospital mortality was defined as death occurring during hospitalisation for AP.

### Outcomes

The study’s primary endpoints were Intensive Care Unit (ICU) admission and in-hospital mortality. In addition, the following clinical outcomes were assessed, as defined above: organ failure (renal, respiratory, haemodynamic) during the hospital admission; the need for endoscopic retrograde cholangiopancreatography (ERCP) and endoscopic sphincterotomy (ES) and its timing; step-up endoscopic drainage of IPN; percutaneous drainage/minimally invasive necrosectomy; open surgical necrosectomy and its timing (early < 2 weeks from the onset of symptoms or late > 4 weeks); the setting of surgical necrosectomy (upfront, or after step-up approach attempts).

### Variables of interest

For each patient, the following variables were analysed retrospectively to find possible associations between IPN and mortality risk or ICU admission.

1. Demographic data and baseline characteristics: sex, age, COVID-19 status on admission, previous episodes of biliary AP, CCI, BMI, clinical history of diabetes, chronic pulmonary disease, arterial hypertension, atrial fibrillation, ischaemic heart disease, chronic kidney disease, diseases of the haematopoietic system, immunosuppressive medications;

2. Clinical risk scores calculated within 72 h from hospital admission: quick Sequential Organ Failure Assessment score (qSOFA), Bedside Index for Severity in Acute Pancreatitis (BISAP), Glasgow-Imrie, Ranson, Acute Physiology and Chronic Health Evaluation II (APACHE II);

3. Stage of the AP according to RAC, and systemic organ complications, including single or multiple organ failure within 72 h from hospital admission (haemodynamic, renal, respiratory);

4. Vital parameters: temperature, systolic blood pressure, heart rate, respiratory rate, and blood oxygen saturation;

5. Laboratory data: white blood cell (WBC) count, neutrophils, platelets, international normalised ratio (INR), CRP, aspartate aminotransferase (AST), alanine aminotransferase (ALT), bilirubin, serum amylase, serum lipase, lactate dehydrogenase (LDH), procalcitonin, lactate;

6. Abdominal findings: diffuse abdominal pain, diffuse abdominal rigidity, localised abdominal pain, localised abdominal rigidity;

7. Concomitant findings: choledocholithiasis, acute cholangitis, timing and type of interventional procedures (ERCP/ES, endoscopic drainage of pancreatic necrosis, percutaneous drainage and minimally invasive necrosectomy, open surgical necrosectomy);

8. Occurrence of complications: ACS, bleeding, bowel fistula, and necrotising cholecystitis;

9. Type of supportive care: antibiotic therapy, antifungal therapy, and nutritional support.

### Statistical analysis

Baseline characteristics of the study population were expressed as absolute numbers and relative frequency measurements for qualitative variables, whereas mean and standard deviation (SD) or the median and standard error (SE)/Interquartile Range (IQR) were used for the quantitative variables. The differences between groups for qualitative variables were determined using the *X*^2^ test (with the Yates correction, when necessary) or Fisher’s exact test as appropriate. Comparisons of quantitative variables between the two groups (survivors and non-survivors or patients admitted and non-admitted to ICU) were performed using the Student *t*-test for variables with parametric distribution and the Mann–Whitney *U* test for those with a non-parametric distribution. Univariable and multivariable logistic regression models were used to identify prognostic factors of mortality and ICU admission. Variables yielding *p* values < 0.05 by univariable analysis and clinical predictors for mortality and complications selected from relevant literature [5, 12, 28] were added to a stepwise prediction model according to their predictive value, indicated by pseudo *R*^2^ (Negelkerke’s *R*^2^ and Cox & Snell *R*^2^) until no further improvement of the model was achieved. The strength of association between a risk factor identified in univariable and multivariable analyses for mortality and ICU admission was determined by calculating odds ratios (OR) and adjusted odds ratios (aOR) with 95% confidence intervals (95% CI). Youden’s J statistic was calculated to identify the optimal cut-point value of laboratory tests. To test model quality and its predictive performance, we plotted the receiver operating characteristics (ROC) curve and computed the area under the curve (AUROC) for the predictive models of mortality and ICU admission. A p value < 0.05 (two-tailed) was considered statistically significant. All the statistical analyses were performed using the Statistical Product and Service Solution (SPSS) 26.0 software (IBM SPSS Statistics, I.B.M. Corp., Armonk, NY, U.S.A.) and Jamovi Computer Software (The Jamovi project (2022). Jamovi (Version 2.3). Retrieved from https://www.jamovi.org).

## Results

### General characteristics of the cohort of patients

**Fig. 1:**
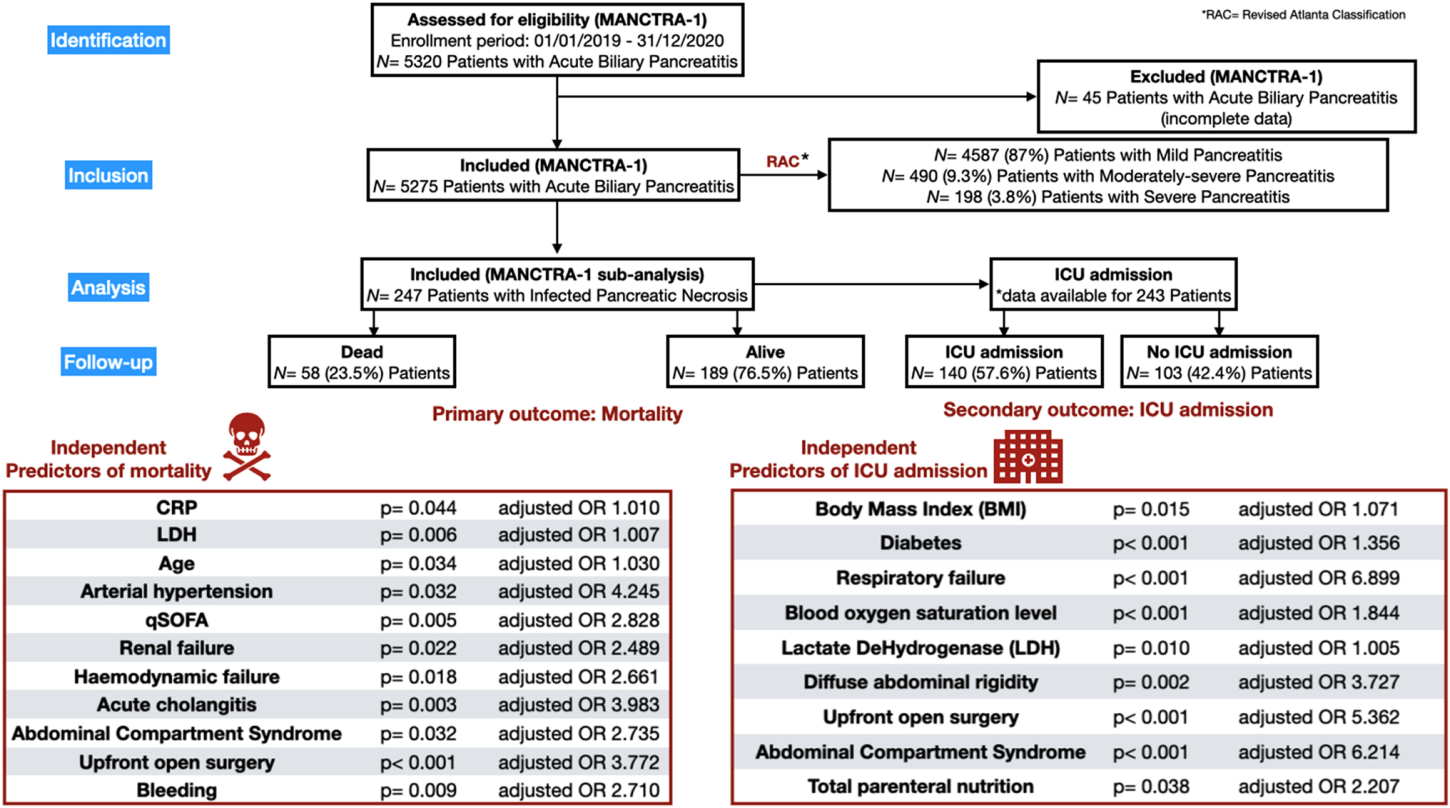
STROBE (Strengthening the Reporting of Observational Studies in Epidemiology) Flow-Diagram

Over the two-year study period (January 2019–December 2020), a total of 5275 patients were included in the MANCTRA-1 database as they were admitted to any of the 150 participating general surgery, hepato-pancreato-biliary (HPB) surgery, gastroenterology or internal medicine departments for biliary AP; 4587 (87%) patients had mild AP, 490 (9.3%) patients had moderately severe AP, and 198 patients had severe AP (3.8%) according to the RAC determined within 72 h from the hospital admission [24]. Figure 1 is the study flowchart. Over the same study period, 247 patients who developed IPN during the hospital stay met the inclusion criteria and were considered for the final post hoc analysis on IPN (Table 1).

**Table 1:** General characteristics of the cohort of patients with infected pancreatic necrosis

Sample size (N. Patients)	247	Number (%)—Mean ± Standard Deviation and Median IQR
Sex (*N*. %)	Male	135 (54.7)
	Female	112 (45.3)
Age (Years)		59.2 ± 17.1; 61 IQR 25.0
COVID-19 Status on admission (*N*. %)	Negative	231 (93.5)
	Positive	16 (6.5)
Previous episodes of biliary pancreatitis (*N*. %)	Yes	83 (35.8)
	No	164 (64.2)
Admitting speciality (*N*. %)	HPB Surgery	43 (17.4)
	Gastroenterology	58 (23.5)
	General Surgery	116 (47.0)
	Internal Medicine	30 (12.1)
Setting of acquisition (*N*. %)	Community	219 (89.3)
	Hospital	28 (10.7)
Charlson’s Comorbidity Index		2.94 ± 3.08; 2 IQR 3
Body Mass Index (BMI) Kg/m^2^		27.5 ± 5.98; 27 IQR 8
Clinical history of diabetes (*N*. %)	Diabetes with organ disfunction	11 (4.5)
	Diabetes without organ disfunction	50 (20.2)
	No diabetes	186 (75.3)
Clinical history of chronic pulmonary disease (*N*. %)	Yes	36 (14.6)
	No	211 (85.4)
Clinical history of hypertension (*N*. %)	Yes	131 (53.0)
	No	116 (47.0)
Clinical history of atrial fibrillation (*N*. %)	Yes	29 (11.7)
	No	218 (88.3)
Clinical history of ischaemic heart disease (*N*. %)	Yes	27 (10.9)
	No	220 (89.1)
Clinical history of chronic kidney disease (*N*. %)	Yes—in permanent replacement therapy	1 (0.4)
	Yes—under medications	13 (5.3)
	No	233 (94.3)
Clinical history of diseases of the hematopoietic system (*N*. %)	Yes	6 (2.4)
	No	241 (97.6)
Patient on immunosuppressive medications (*N*. %)	Yes	10 (4.0)
	No	237 (96.0)
qSOFA		0.966 ± 1.03; 1 IQR 2.00
BISAP (Bedside Index of Severity in Acute Pancreatitis) score		2.10 ± 1.50; 2.00 IQR 2.00
Glasgow-Imrie criteria		2.85 ± 1.62; 3.00 IQR 2.00
Ranson’s criteria		2.93 ± 1.62; 3.00 IQR 2.00
APACHE II score		8.06 ± 5.24; 7.00 IQR 5.00
Revised Atlanta Classification (RAC) stage (*N*. %)	Mild acute pancreatitis	88 (35.6)
	Moderately-severe acute pancreatitis	67 (27.1)
	Severe acute pancreatitis	92 (37.2)
Organ failure during the hospital admission (*N*. %)	None	93 (37.7)
	Haemodynamic	26 (10.5)
	Haemodynamic—renal	5 (2.0)
	Haemodynamic—respiratory	9 (3.6)
	Haemodynamic—respiratory—renal	16 (6.5)
	Renal	41 (16.6)
	Respiratory	46 (18.6)
	Respiratory—renal	11 (4.5)
Temperature on admission °C		36.9 ± 1.36; 36.9 IQR 1.20
Systolic blood pressure on admission (mmHg)		125 ± 45.1; 120 IQR 34.00
Heart rate on admission (bpm)		92.1 ± 18.5; 90.0 IQR 26.00
Respiratory rate on admission (breaths/min)		19.0 ± 4.13; 18.0 IQR 6.00
Blood oxygen saturation level (SpO_2_%) on admission		95.1 ± 3.81; 96.0 IQR 4.00
WBC on admission (cells/mm^3^)		16.9 ± 6.5; 17.0 IQR 7.98
Neutrophils on admission (cells/mm^3^)		14.1 ± 6.10; 14.1 IQR 7.90
Platelets on admission (mcL)		267 ± 129; 247 IQR 157
INR—International Normalised Ratio on admission		1.37 ± 0.654; 1.20 IQR 0.407
CRP—C-reactive Protein on admission (mg/L)		121 ± 125; 71.0 IQR 181
AST—Aspartate aminotransferase on admission (U/L)		180 ± 184; 104 IQR 217
ALT—Alanine aminotransferase on admission (U/L)		215 ± 286; 103 IQR 232
Total Bilirubin on admission (mg/dL)		2.65 ± 2.64; 1.60 IQR 2.41
Conjugated Bilirubin on admission (mg/dL)		1.54 ± 1.65; 0.910 IQR 1.60
Serum Amylase on admission (U/L)		1463 ± 1440; 901 IQR 1702
Serum Lipase on admission (U/L)		2870 ± 3540; 1270 IQR 3700
LDH—Lactate DeHydrogenase on admission (U/L)		531 ± 510; 410 IQR 314
Procalcitonin on admission (*N*g/mL)		3.78 ± 6.40; 1.66 IQR 3.60
Lactates on admission (mmol/L)		2.65 ± 1.54; 2.25 IQR 1.92
Abdominal findings (*N*. %)	Diffuse abdominal pain	99 (40.1)
	Diffuse abdominal rigidity	30 (12.1)
	Localised abdominal pain	91 (36.8)
	Localised abdominal rigidity	20 (8.1)
	No abdominal pain/no abdominal rigidity	7 (2.8)
Concomitant choledocholithiasis (*N*. %)	No	164 (66.4)
	Yes	59 (23.9)
	Yes, with common bile duct obstruction	24 (9.7)
Concomitant cholangitis (*N*. %)	Yes	36 (14.6)
	No	211 (85.4)
ERCP/ES (*N*. %)	No	187 (75.7)
	Yes, within 24 h from hospital admission	8 (3.2)
	Yes, between 24–48 h from hospital admission	18 (7.3)
	Yes, between 48–72 h from hospital admission	19 (7.7)
	Yes, > 72 h from hospital admission	15 (6.1)
Endoscopic step-up drainage of pancreatic necrosis (*N*. %)	Yes	56 (22.7)
	No	191 (77.3)
Surgical necrosectomy (*N*. %)	No	162 (65.9)
	Yes, minimally-invasive	63 (25.6)
	Yes, open	22 (8.5)
Timing of surgical necrosectomy (*N*. %)	< 2 weeks from the onset of symptoms	27 (32.0)
	2–4 weeks from the onset of symptoms	28 (32.0)
	> 4 weeks from the onset of symptoms	30 (36.0)
Setting of surgical necrosectomy (*N*. %)	Upfront	54 (61.3)
	After failure of endoscopic necrosectomy attempt	8 (8.6)
	After failure of percutaneous and endoscopic necrosectomy attempt	23 (30.1)
Abdominal compartment syndrome (*N*. %)	Yes	28 (11.3)
	No	219 (88.7)
Bleeding (*N*. %)	Yes	44 (17.8)
	No	203 (82.2)
Bowel fistula (*N*. %)	Yes	21 (8.5)
	No	226 (91.5)
Necrotizing cholecystitis (*N*. %)	Yes	18 (7.3)
	No	229 (92.7)
Antibiotic therapy (*N*. %)	Yes	212 (85.8)
	No	35 (14.2)
Antifungal therapy (*N*. %)	Yes	91 (36.8)
	No	156 (63.2)
Nutritional support (*N*. %)	Nihil per os	63 (25.5)
	Total parenteral nutrition	85 (34.4)
	Oral	47 (19.0)
	Enteral via naso-gastric tube	31 (12.5)
	Enteral via naso-jejunal tube	21 (8.5)
ICU admission (*N*. %)	Yes	140 (57.6)
	No	103 (42.4)
Mortality (*N*. %)	Yes	58 (23.5%)
	No	189 (76.5%)

*HPB* Hepato-pancreato-biliary, *RAC* Revised Atlanta Classification, *qSOFA* quick Sepsis-related Organ Failure Assessment, *BISAP* Bedside Index of Severity in Acute Pancreatitis, *APACHE II* Acute Physiology, Age, and Chronic Health Evaluation II, *ERCP/ES* Endoscopic Retrograde Cholangio-Pancreatography/Endoscopic Sphincterotomy

### Predictors of ICU admission

The statistics for this outcome were performed on 243 patients (missed data for four patients, 1.6%).

The univariable analysis demonstrated a significant association between several demographic factors and the risk of ICU admission during the hospitalisation for IPN (Table 2). Mean BMI was higher in patients admitted to ICU than those who did not need ICU support (*p* = 0.015; MD 2.371). Similarly, diabetes was more common in patients admitted to ICU (*p* < 0.001; OR 1.321). Looking at the predictive scores, patients admitted to ICU had higher values of qSOFA (*p* < 0.001; MD 0.625), BISAP (*p* = 0.021; MD 0.644), Glasgow-Imrie (*p* < 0.001; MD 1.137), Ranson’s (*p* < 0.001; MD 1.145), and APACHE II (*p* = 0.035; MD 2.247). A higher rate of patients with severe AP was found in patients admitted to ICU (*p* < 0.001; OR 7.137) and organ failure was more common in patients who needed ICU admission compared with those who did not (*p* < 0.001; OR 10.343). Respiratory failure showed the strongest association (*p* < 0.001; OR 10.765), followed by haemodynamic (*p* < 0.001; OR 3.713) and renal failure (*p* < 0.001; OR 3.187). Heart rate on admission was higher in patients admitted to ICU (*p* = 0.008; MD 6.374), whereas the mean blood oxygen saturation level was lower (*p* < 0.001; MD 2.001). On laboratory tests, patients admitted to ICU showed higher WBC count (*p* = 0.003; MD 1.874), CRP levels (*p* = 0.049; MD 34.423), LDH (p = 0.010; MD 252) and lactate (*p* = 0.036; MD 0.610).

**Table 2:** Results of the univariable and multivariable analyses. Outcome intensive care unit (ICU) admission

Predictor ICU admission (*N*. Patients, %)	Yes = 140 (57.6)	No = 103 (42.4)	Odds ratio (OR) or Mean difference (MD)	95% CI	*p*-value		adjusted OR (aOR)	95% CI	*p*-value
Sex (*N*. %)									
Female	65 (46.4)	45 (43.6)	OR = 0.895	[0.537; 1.492]	0.672				
Male	75 (53.6)	58 (56.4)							
Age (Years) Mean ± SD (Median; SE)	58.6 ± 15.4 (60.5; 1.4)	60.4 ± 19.2 (61.0; 1.89)	MD = 1.851	[– 2.53; 1.231]	0.407				
COVID-19 Status on admission (*N*. %)									
Negative	129 (92.1)	97 (94.2)	OR = 1.383	[0.493; 3.863]	0.539				
Positive	11 (7.9)	6 (5.8)							
Previous episodes of biliary pancreatitis (*N*. %)									
No	93 (66.4)	67 (65.0)	OR = 0.941	[0.550:1.612]	0.823				
Yes	47 (33.6)	36 (35.0)							
Admitting speciality (*N*. %)									
HPB Surgery	27 (19.3)	16 (15.5)	OR = 1.302	[0.659; 2.561]	0.449				
Other	113 (80.7)	87 (84.5)							
Charlson’s Comorbidity Index Mean ± SD (Median; SE)	3.05 ± 3.98 (2.00; 0.337)	2.97 ± 2.60 (3.00; 0.256)	MD = 0.079	[– 0.965; 0.807]	0.649				
Body Mass Index (BMI) Kg/m^2^ Mean ± SD (Median; SE)	28.2 ± 5.88 (27.1; 0.613)	25.9 ± 5.71 (25.7; 0.737)	MD = 2.371	[– 4.281; – 0.467]	0.015		1.071	[1.004; 1.143]	0.035
Clinical history of diabetes (*N*. %)									
No	94 (67.1)	89 (86.4)	OR = 1.321	[1.165; 1.625]	< 0.001		1.356	[1.150; 1.841]	0.018
Yes	46 (32.9)	14 (13.6)							
Clinical history of chronic pulmonary disease (*N*. %)									
No	115 (82.1)	92 (89.3)	OR = 1.823	[0.850; 3.892]	0.120				
Yes	25 (17.9)	11 (10.7)							
Clinical history of hypertension (*N*. %)									
No	66 (47.1)	49 (47.6)	OR = 1.021	[0.611; 1.694]	0.947				
Yes	74 (52.9)	54 (52.4)							
Clinical history of atrial fibrillation (*N*. %)									
No	121 (86.4)	93 (90.3)	OR = 1.463	[0.648; 3.291]	0.359				
Yes	19 (13.6)	10 (9.7)							
Clinical history of ischaemic heart disease (*N*. %)									
No	123 (87.8)	95 (92.2)	OR = 1.642	[0.679; 3.962]	0.267				
Yes	17 (12.2)	8 (7.8)							
Clinical history of chronic kidney disease (*N*. %)									
No	133 (95.0)	96 (93.2)	OR = 0.722	[0.245; 2.137]	0.533				
Yes	7 (5.0)	7 (6.8)							
Clinical history of diseases of the hematopoietic system (*N*. %)									
No	138 (98.6)	99 (96.1)	OR = 0.359	[0.064; 2.004]	0.223				
Yes	2 (1.4)	4 (3.9)							
Patient on immunosuppressive medications (*N*. %)									
No	135 (96.4)	98 (95.1)	OR = 0.726	[0.205; 2.587]	0.619				
Yes	5 (3.6)	5 (4.9)							
qSOFA Mean ± SD (Median; SE)	1.21 ± 1.03 (1.00; 0.106)	0.588 ± 0.920 (0.00; 0.129)	MD = 0.625	[– 0.965; – 0.284]	< 0.001		1.550	[0.762; 3.152]	0.226
BISAP score Mean ± SD (Median; SE)	2.35 ± 1.53 (2.00; 0.165)	1.70 ± 1.39 (2.00; 0.210)	MD = 0.644	[– 1.197; – 0.099]	0.021		0.760	[0.460; 1.254]	0.283
Glasgow-Imrie criteria Mean ± SD (Median; SE)	3.30 ± 1.52 (3.00; 0.169)	2.16 ± 1.53 (2.00; 0.233)	MD = 1.137	[– 1.703; – 0.565]	< 0.001		1.290	[0.722; 2.303]	0.389
Ranson’s criteria Mean ± SD (Median; SE)	3.36 ± 1.56 (3.00; 0.167)	2.21 ± 1.46 (2.00; 0.225)	MD = 1.145	[– 1.715; – 0.575]	< 0.001		1.476	[0.875; 2.489]	0.144
APACHE II score Mean ± SD (Median; SE)	9.11 ± 5.95 (7.00; 0.756)	6.87 ± 3.37 (7.00; 0.540)	MD = 2.247	[– 4.323; – 0.165]	0.035		1.096	[0.953; 1.260]	0.198
Revised Atlanta Classification (RAC) stage (*N*. %)									
Moderately severe	45 (32.1)	22 (21.3)	OR = 1.745	[0.967; 3.153]	0.063				
Severe	74 (52.8)	14 (13.6)	OR = 7.137	[3.712; 13.735]	< 0.001		4.464	[1.061; 18.787]	0.041
Organ failure during the hospital admission (*N*. %)									
No	34 (24.3)	69 (66.9)	OR = 10.343	[5.631; 18.903]	< 0.001		0.835	[0.139; 5.020]	0.844
Yes	106 (75.7)	34 (33.1)							
Renal failure during the hospital admission (*N*. %)									
No	86 (61.4)	86 (83.5)	OR = 3.187	[1.712; 5.916]	< 0.001		2.380	[0.676; 8.383]	0.177
Yes	54 (38.6)	17 (16.5)							
Haemodynamic failure during the hospital admission (*N*. %)									
No	97 (69.3)	92 (89.3)	OR = 3.713	[1.801; 7.634]	< 0.001		2.267	[0.645; 7.972]	p = 0.202
Yes	43 (30.7)	11 (10.7)							
Respiratory failure during the hospital admission (N. %)									
No	69 (49.3)	94 (91.3)	OR = 10.765	[5.031; 23.076]	< 0.001		6.899	[1.951; 24.396]	0.003
Yes	71 (50.7)	9 (8.7)							
Temperature on admission °C Mean ± SD (Median; SE)	37.0 ± 1.66 (37.0; 0.141)	36.8 ± 0.812 (36.6; 0.0800)	MD = 0.196	[– 0.545; 0.154]	0.271				
Systolic blood pressure on admission (mmHg) Mean ± SD (Median; SE)	121 ± 56.6 (110; 4.78)	129 ± 22.0 (128; 2.17)	MD = 7.837	[– 3.764; 19.454]	0.184				
Heart rate on admission (bpm) Mean ± SD (Median; SE)	94.8 ± 18.3 (95.0; 1.55)	88.4 ± 18.7 (87.0; 1.84)	MD = 6.374	[– 11.143; – 1.614]	0.008		1.013	[0.997; 1.028]	0.113
Respiratory rate on admission (breaths/min) Mean ± SD (Median; SE)	19.8 ± 4.68 (19.0; 0.397)	18.7 ± 6.87 (18.0; 0.680)	MD = 1.087	[– 2.556; 0.385]	0.148				
Blood oxygen saturation level (SpO_2_%) on admission Mean ± SD (Median; SE)	94.2 ± 4.45 (95.0; 0.378)	96.2 ± 2.36 (96.0; 0.234)	MD = 2.001	[1.053; 2.964]	< 0.001		1.844	[1.764; 1.931]	< 0.001
WBC on admission (cells/mm^3^) Mean ± SD (Median; SE)	17.8 ± 6.35 (18.0; 0.548)	15.9 ± 6.62 (15.7; 0.676)	MD = 1.874	[– 3.576; – 0.168]	0.031		1.226	[0.861; 1.749]	0.258
Neutrophils on admission (cells/mm^3^) Mean ± SD (Median; SE)	14.8 ± 6.41 (14.5; 0.590)	13.4 ± 5.67 (13.1; 0.588)	MD = 1.393	[– 3.063; 0.276]	0.101				
Platelets on admission (mcL) Mean ± SD (Median; SE)	268 ± 136 (252; 11.8)	265 ± 123 (237; 12.5)	MD = 3.704	[– 38.216; 30.810]	0.833				
INR—International Normalised Ratio on admission Mean ± SD (Median; SE)	1.39 ± 0.637 (1.20; 0.0582)	1.33 ± 0.693 (1.14; 0.0765)	MD = 0.677	[– 0.254; 0.119]	0.476				
CRP—C-reactive Protein on admission (mg/L) Mean ± SD (Median; SE)	130 ± 131 (85.7; 12.5)	95.9 ± 103 (49.0; 11.3)	MD = 34.423	[– 68.712; – 0.153]	0.049		1.006	[0.999; 1.014]	0.085
AST—Aspartate aminotransferase on admission (U/L) Mean ± SD (Median; SE)	189 ± 187 (116.0; 17.7)	168 ± 183 (84.0; 21.2)	MD = 21.134	[– 75.712; 33.532]	0.446				
ALT—Alanine aminotransferase on admission (U/L) Mean ± SD (Median; SE)	216 ± 280 (110; 25.1)	218 ± 299 (92.5; 30.5)	MD = 2.224	[– 74.923; 79.411]	0.955				
Total Bilirubin on admission (mg/dL) Mean ± SD (Median; SE)	2.93 ± 2.85 (2.00; 0.250)	2.30 ± 2.34 (1.46; 0.239)	MD = 0.636	[– 1.341; 0.064]	0.075				
Conjugated Bilirubin on admission (mg/dL) Mean ± SD (Median; SE)	1.60 ± 1.74 (0.900; 0.177)	1.46 ± 1.54 (0.920; 0.211)	MD = 0.140	[– 0.705; 0.424]	0.624				
GGT—Gamma-Glutamyl Transpeptidase on admission (U/L) Mean ± SD (Median; SE)	287 ± 301 (197; 32.8)	217 ± 236 (116; 32.1)	MD = 70.010	[– 166.621; 25.634]	0.150				
Serum Amylase on admission (U/L) Mean ± SD (Median; SE)	1490 ± 1531 (829; 149)	2621 ± 5196 (983; 596)	MD = 1131.103	[0.017; 0.616]	0.036		0.997	[0.993; 1.000]	0.056
Serum Lipase on admission (U/L) Mean ± SD (Median; SE)	3907 ± 6652 (1098; 701)	4920 ± 6714 (2105; 814)	MD = 1013.103	[– 1107–3133]	0.347				
LDH—Lactate DeHydrogenase on admission (U/L) Mean ± SD (Median; SE)	625 ± 606 (476; 67.4)	373 ± 183 (313; 28.2)	MD = 252.112	[– 441; – 61.7]	0.010		1.005	[1.001; 1.009]	0.012
Procalcitonin on admission (ng/mL) Mean ± SD (Median; SE)	4.50 ± 6.53 (2.95; 0.932)	2.38 ± 6.12 (0.400; 1.28)	MD = 2.131	[– 5.351; 1.102]	0.193				
Lactates on admission (mmol/L) Mean ± SD (Median; SE)	2.89 ± 1.60 (2.70; 0.193)	2.28 ± 1.37 (1.90; 0.200)	MD = 0.610	[– 1.183; – 0.041]	0.036		1.470	[0.877; 2.463]	0.143
Diffuse abdominal pain (*N*. %)									
No	85 (60.7)	62 (60.2)	OR = 1.023	[0.607; 1.724]	0.935				
Yes	55 (39.3)	41 (39.8)							
Diffuse abdominal rigidity (*N*. %)									
No	115 (82.1)	98 (95.1)	OR = 1.235	[1.086; 1.636]	0.002		3.727	[1.315; 10.560]	0.013
Yes	25 (17.9)	5 (4.9)							
Concomitant choledocholithiasis (*N*. %)									
No	90 (64.3)	72 (69.9)	OR = 1.291	[0.748; 2.223]	0.359				
Yes	50 (35.7)	31 (30.1)							
Concomitant common bile duct obstruction (*N*. %)									
No	121 (86.4)	98 (95.1)	OR = 3.083	[1.112; 8.541]	0.024		2.346	[0.733; 7.120]	0.132
Yes	19 (13.6)	5 (4.9)							
Concomitant acute cholangitis (*N*. %)									
No	114 (81.4)	93 (90.3)	OR = 2.124	[0.974; 4.621]	0.055		1.692	[0.717; 3.990]	0.230
Yes	26 (18.6)	10 (9.7)							
ERCP/ES > 48 h for concomitant choledocholithiasis, common bile duct obstruction, or cholangitis (*N*. %)									
No	29 (49.1)	27 (75.0)	OR = 3.104	[1.253; 7.721]	0.018		4.250	[1.190; 15.180]	0.026
Yes	30 (50.9)	9 (25.0)							
ERCP/ES ≤ 48 h for concomitant choledocholithiasis, common bile duct obstruction, or cholangitis (*N*. %)									
No	40 (67.8)	30 (83.3)	OR = 2.382	[0.846; 6.673]	0.149				
Yes	19 (32.2)	6 (16.7)							
Endoscopic step-up drainage of pancreatic necrosis (*N*. %)									
No	101 (72.1)	86 (83.5)	OR = 1.953	[1.031; 3.702]	0.045		1.755	[0.319; 9.670]	0.518
Yes	39 (27.9)	17 (16.5)							
Upfront open surgical necrosectomy (*N*. %)									
No	85 (60.7)	98 (95.1)	OR = 12.734	[4.852; 33.110]	< 0.001		5.362	[1.199; 23.990]	0.028
Yes	55 (39.3)	5 (4.9)							
Percutaneous drainage/minimally invasive necrosectomy (*N*. %)									
No	125 (89.3)	97 (94.2)	OR = 1.941	[0.726; 5.193]	0.248				
Yes	15 (10.7)	6 (5.8)							
Surgical necrosectomy < 2 weeks from the onset (*N*. %)									
No	49 (66.2)	16 (88.9)	OR = 4.001	[0.847; 18.902]	0.081				
Yes	25 (33.8)	2 (11.1)							
Surgical necrosectomy 2–4 weeks from the onset (*N*. %)									
No	22 (52.4)	11 (57.9)	OR = 2.853	[0.944; 8.601]	0.074				
Yes	20 (47.6)	8 (42.1)							
Surgical necrosectomy > 4 weeks from the onset (*N*. %)									
No	48 (66.7)	12 (66.6)	OR = 1.012	[0.331; 3.063]	0.990				
Yes	24 (33.3)	6 (33.4)							
Upfront surgical necrosectomy (*N*. %)									
No	29 (38.2)	7 (50.0)	OR = 1.591	[0.504; 4.992]	0.428				
Yes	47 (61.8)	7 (50.0)							
Abdominal compartment syndrome (*N*. %)									
No	114 (81.4)	101 (98.1)	OR = 11.534	[2.672; 49.713]	< 0.001		6.214	[1.356; 28.490]	0.019
Yes	26 (18.6)	2 (1.9)							
Bleeding (*N*. %)									
No	104 (74.3)	96 (93.2)	OR = 4.754	[2.021; 11.234]	< 0.001		3.357	[1.373; 8.210]	0.008
Yes	36 (25.7)	7 (6.8)							
Bowel fistula (*N*. %)									
No	122 (87.1)	100 (87.1)	OR = 4.922	[1.413; 17.211]	0.006		2.009	[0.504; 8.000]	0.323
Yes	18 (12.9)	3 (12.9)							
Necrotizing cholecystitis (*N*. %)									
No	124 (88.6)	101 (98.1)	OR = 6.524	[1.461; 29.034]	0.005		2.794	[0.551; 14.160]	0.215
Yes	16 (11.4)	2 (1.9)							
Antibiotic therapy (*N*. %)									
No	13 (9.3)	22 (21.3)	OR = 2.651	[1.273; 5.564]	0.010		1.400	[0.630; 3.111]	0.408
Yes	127 (90.7)	81 (78.7)							
Antifungal therapy (*N*. %)									
No	70 (50.0)	83 (80.6)	OR = 4.153	[2.302; 7.491]	< 0.001		3.565	[1.920; 6.620]	< 0.001
Yes	70 (50.0)	20 (19.4)							
Total Parenteral Nutrition (*N*. %)									
No	84 (60.0)	75 (78.2)	OR = 1.793	[1.034; 3.101]	0.038		2.207	[1.067; 4.568]	0.033
Yes	56 (40.0)	28 (21.8)							
Enteral nutrition (*N*. %)									
No	81 (57.8)	65 (63.1)	OR = 0.803	[0.476; 1.353]	0.429		0.487	[0.245; 0.967]	0.040
Yes	59 (42.2)	38 (36.9)							

*RAC *Revised Atlanta Classification, *qSOFA *quick Sepsis-related Organ Failure Assessment, *BISAP *Bedside Index of Severity in Acute Pancreatitis*, APACHE II *Acute Physiology, Age, and Chronic Health Evaluation II, *ERCP/ES *Endoscopic Retrograde Cholangio-Pancreatography/Endoscopic Sphincterotomy, *WBC *white blood cells

Patients admitted to ICU more commonly had diffuse abdominal rigidity on hospital admission (*p* = 0.002; OR 1.235) and concomitant common bile duct obstruction (*p* = 0.024; OR 3.083). Patients with an indication for ERCP/ES (choledocholithiasis, common bile duct obstruction, cholangitis) were at increased risk of ICU admission if the procedure was performed later than 48 h (*p* = 0.018; OR 3.104) from hospital admission. Open surgical necrosectomy was associated with a higher risk of ICU admission (*p* < 0.001; OR 12.734).

Among the analysed complications of AP, ACS (p < 0.001; OR 11.534) was associated with the risk of ICU admission on the univariable analysis, followed by necrotising cholecystitis (*p* = 0.005; OR 6.524), bowel fistula (*p* = 0.006; OR 4.922), and bleeding (*p* < 0.001; OR 4.754). Total parenteral nutrition (*p* = 0.038; OR 1.793), but not enteral nutrition (p = 0.429; OR 0.803), was associated with a higher risk of ICU admission.

In the multivariable analysis, BMI (*p* = 0.035; aOR 1.071), diabetes (p = 0.018; aOR 1.356), severe AP (*p* = 0.041; aOR 4.464), respiratory failure (*p* = 0.003; aOR 6.899), blood oxygen saturation (*p* < 0.001; aOR 1.844), LDH (*p* = 0.012; aOR 1.005), diffuse abdominal rigidity (*p* = 0.013; aOR 3.727), upfront open surgical necrosectomy (*p* = 0.028; aOR 5.362), ERCP/ES performed > 48 h from hospital admission (*p* = 0.026, aOR 4.250), and total parenteral nutrition (p = 0.033; aOR 2.207) were independent predictors of ICU admission. Enteral feeding (*p* = 0.040; aOR 0.487) was shown to be a protective factor against the risk of ICU admission (Table 3).

**Table 3:** Results of the univariable and multivariable analyses. Outcome mortality

Predictor in-hospital mortality (*N*. Patients, %)	Yes = 58 (23.5)	No = 189 (76.5)	Odds Ratio (OR) or Mean Difference (MD)	95% CI	*p*-value	Adjusted OR (aOR)	95% CI	*p*-value
Sex (*N*. %)								
Male	30 (51.7)	105 (55.5)	OR = 0.852	[0.471; 1.552]	0.652			
Female	28 (48.3)	84 (44.5)						
Age (Years) Mean ± SD (Median; SE)	63.1 ± 16.3 (63.5; 2.15)	58.0 ± 17.2 (60.0; 1.25)	MD = 5.001	[10.023; – 4.351]	0.051	1.030	[1.002; 1.158]	0.034
COVID-19 Status on admission (*N*. %)								
Negative	52 (89.6)	179 (94.7)	OR = 2.073	[0.711; 5.953]	0.219			
Positive	6 (10.4)	10 (5.3)						
Previous episodes of biliary pancreatitis (*N*. %)								
No	36 (62.1)	117 (59.8)	OR = 0.750	[0.383; 1.451]	0.417			
Yes	22 (37.9)	72 (40.2)						
Admitting speciality (*N*. %)								
HPB Surgery	5 (8.6)	38 (20.1)	OR = 0.375	[0.140; 0.999]	0.044	0.164	[0.025; 1.052]	0.057
Other	53 (91.4)	151 (79.9)						
Setting of acquisition (N. %)								
Community acquired	50 (84.4)	170 (88.8)	OR = 1.525	[0.621; 3.721]	0.337			
Hospital acquired	8 (15.6)	19 (11.2)						
Charlson’s Comorbidity Index Mean ± SD (Median; SE)	3.97 ± 4.55 (3.00; 0.59)	2.62 ± 2.40 (2.00; 0.17)	MD = 1.003	[– 2.002; – 4.261]	0.021	1.781	[1.505; 2.210]	0.269
Body Mass Index (BMI) Kg/m^2^ Mean ± SD (Median; SE)	29.48 ± 6.12 (28.85; 0.94)	26.76 ± 5.79 (26.20; 0.54)	MD = 2.701	[– 4.803; – 0.801]	0.012	1.057	[0.976; 1.145]	0.172
Clinical history of diabetes (*N*. %)								
No	36 (62.1)	150 (79.3)	OR = 1.425	[1.222; 1.801]	0.009	0.717	[0.226; 2.272]	0.572
Yes	22 (37.9)	39 (20.7)						
Clinical history of chronic pulmonary disease (*N*. %)								
No	46 (79.3)	165 (87.3)	OR = 1.796	[0.836; 3.861]	0.140			
Yes	12 (20.7)	24 (12.7)						
Clinical history of hypertension (*N*. %)								
No	16 (27.6)	100 (52.9)	OR = 2.954	[1.553; 5.612]	< 0.001	4.245	[1.135; 15.882]	0.032
Yes	42 (72.4)	89 (47.1)						
Clinical history of atrial fibrillation (*N*. %)								
No	51 (87.9)	167 (88.3)	OR = 1.047	[0.421; 2.581]	0.929			
Yes	7 (12.1)	22 (11.7)						
Clinical history of ischaemic heart disease (*N*. %)								
No	50 (86.2)	170 (89.9)	OR = 1.434	[0.593; 3.473]	0.471			
Yes	8 (13.8)	19 (10.1)						
Clinical history of chronic kidney disease (*N*. %)								
No	52 (89.6)	181 (95.7)	OR = 2.615	[0.862; 7.865]	0.078			
Yes	6 (10.4)	8 (4.3)						
Clinical history of diseases of the hematopoietic system (N. %)								
No	57 (98.3)	184 (97.3)	OR = 0.646	[0.071; 5.645]	0.690			
Yes	1 (1.7)	5 (2.7)						
Patient on immunosuppressive medications (*N*. %)								
No	57 (98.3)	180 (95.2)	OR = 0.351	[0.041; 2.831]	0.460			
Yes	1 (1.7)	9 (4.8)						
qSOFA Mean ± SD (Median; SE)	1.36 ± 0.98 (1.00; 0.15)	0.81 ± 1.01 (0.00; 0.09)	MD = 1.003	[– 1.001; – 2.372]	0.002	2.828	[1.359; 5.879]	0.005
BISAP score Mean ± SD (Median; SE)	2.48 ± 1.20 (3.00; 0.18)	1.95 ± 1.59 (2.00; 0.16)	MD = 1.002	[– 1.004; – 5.461]	0.002	0.792	[0.461; 1.360]	0.399
Glasgow-Imrie criteria Mean ± SD (Median; SE)	3.26 ± 1.42 (3.00; 0.24)	2.70 ± 1.67 (2.00; 0.17)	MD = 1.003	[– 1.002; – 3.701]	0.043	1.197	[0.697; 2.056]	0.514
Ranson’s criteria Mean ± SD (Median; SE)	3.33 ± 1.47 (3.00; 0.24)	2.78 ± 1.65 (2.00; 0.16)	MD = 1.004	[– 1.003; 1.202]	0.064			
APACHE II score Mean ± SD (Median; SE)	9.20 ± 5.49 (8.50; 1.00)	7.60 ± 5.10 (7.00; 0.59)	MD = 2.001	[– 3.001; 1.001]	0.175			
Revised Atlanta Classification (RAC) stage (*N*. %)								
Moderately severe	18 (31.1)	49 (25.9)	OR = 1.293	[0.671; 2.452]	0.444			
Severe	34 (58.6)	58 (30.7)	OR = 3.204	[1.742; 5.871]	< 0.001	2.114	[0.595; 7.512]	0.247
APACHE II score Mean ± SD (Median; SE)	9.20 ± 5.49 (8.50; 1.00)	7.60 ± 5.10 (7.00; 0.59)	MD = 2.001	[– 3.001; 1.001]	0.175			
Revised Atlanta Classification (RAC) stage (*N*. %)								
Moderately severe	18 (31.1)	49 (25.9)	OR = 1.293	[0.671; 2.452]	0.444			
Severe	34 (58.6)	58 (30.7)	OR = 3.204	[1.742; 5.871]	< 0.001	2.114	[0.595; 7.512]	0.247
Organ failure during the hospital admission (*N*. %)								
No	4 (6.9)	114 (66.2)	OR = 13.443	[4.653; 38.411]	< 0.001	11.589	[3.873; 34.671]	< 0.001
Yes	54 (93.1)	64 (33.8)						
Renal failure during the hospital admission (*N*. %)								
No	27 (46.5)	146 (77.2)	OR = 3.901	[2.101; 7.231]	< 0.001	2.489	[1.138; 5.442]	0.022
Yes	31 (53.5)	43 (22.8)						
Haemodynamic failure during the hospital admission (*N*. %)								
No	33 (56.9)	158 (83.6)	OR = 3.864	[2.022; 7.371]	< 0.001	2.661	[1.184; 5.978]	0.018
Yes	25 (43.1)	31 (16.4)						
Respiratory failure during the hospital admission (*N*. %)								
No	28 (48.3)	137 (72.5)	OR = 2.823	[1.541; 5.171]	< 0.001	2.033	[0.906; 4.560]	0.085
Yes	30 (51.7)	52 (27.5)						
Temperature on admission °C Mean ± SD (Median; SE)	36.8 ± 2.31 (37.0; 0.30)	36.9 ± 0.89 (36.8; 0.06)	MD = 9.513	[– 0.403; 0.202]	0.656			
Systolic blood pressure on admission (mmHg) Mean ± SD (Median; SE)	117 ± 27.2 (110; 3.57)	127 ± 49.1 (120; 3.57)	MD = 8.004	[– 8.101; 15.023]	0.054			
Heart rate on admission (bpm) Mean ± SD (Median; SE)	95.9 ± 17.8 (97.0; 2.33)	90.9 ± 18.7 (88.5; 1.36)	MD = 5.031	[– 10.522; 0.421]	0.071			
Respiratory rate on admission (breaths/min) Mean ± SD (Median; SE)	20.3 ± 3.74 (20.0; 0.49)	18.6 ± 4.17 (18.0; 0.30)	MD = 2.007	[– 3.001; – 1.001]	0.002	1.078	[0.998; 1.163]	0.056
Blood oxygen saturation level (SpO_2_%) on admission Mean ± SD (Median; SE)	93.9 ± 4.68 (95.0; 0.61)	95.4 ± 3.44 (96.0; 0.25)	MD = 1.003	[1.642; 2.001]	0.019	0.938	[0.869; 1.010]	0.098
WBC on admission (cells/mm^3^) Mean ± SD (Median; SE)	17.7 ± 7.13 (17.5; 0.94)	16.7 ± 6.28 (16.7; 0.47)	MD = 1.071	[– 3.021; 0.881]	0.281			
Neutrophils on admission (cells/mm^3^) Mean ± SD (Median; SE)	14.9 ± 7.03 (14.2; 1.00)	13.8 ± 5.80 (13.7; 0.45)	MD = 0.601	[– 2.503; 1.401]	0.570			
Platelets on admission (mcL) Mean ± SD (Median; SE)	256 ± 137 (229; 18.3)	270 ± 127 (255; 9.59)	MD = 17.045	[– 17.034; 53.012]	0.309			
INR—International Normalised Ratio on admission Mean ± SD (Median; SE)	1.48 ± 0.80 (1.25; 0.10)	1.33 ± 0.58 (1.17; 0.04)	MD = 0.063	[– 0.181; 0.011]	0.122			
CRP—C-reactive Protein on admission (mg/L) Mean ± SD (Median; SE)	126 ± 129 (59.0; 20.0)	119 ± 124 (73.8; 9.96)	MD = 1.401	[– 24.011; 22.321]	0.848	1.010	[1.023; 1.103]	0.044
AST—Aspartate aminotransferase on admission (U/L) Mean ± SD (Median; SE)	199 ± 181 (138; 26.2)	173 ± 185 (101; 15.5)	MD = 18.023	[– 60.023; 14.011]	0.268			
ALT—Alanine aminotransferase on admission (U/L) Mean ± SD (Median; SE)	237 ± 325 (142; 44.6)	208 ± 273 (96.0; 20.8)	MD = 13.012	[– 57.021; 19.032]	0.489			
Total Bilirubin on admission (mg/dL) Mean ± SD (Median; SE)	2.62 ± 2.48 (1.79; 0.33)	2.66 ± 2.69 (1.60; 0.20)	MD = 2.014	[– 0.361; 0.401]	0.959			
Conjugated Bilirubin on admission (mg/dL) Mean ± SD (Median; SE)	1.72 ± 1.94 (0.91; 0.28)	1.47 ± 1.52 (0.91; 0.14)	MD = 4.796	[– 0.302; 0.294]	0.957			
Serum Amylase on admission (U/L) Mean ± SD (Median; SE)	1585 ± 1460 (901; 223)	1426 ± 1437 (905; 121)	MD = 47.034	[– 302; 165]	0.553			
Serum Lipase on admission (U/L) Mean ± SD (Median; SE)	3201 ± 4155 (1499; 683)	2772 ± 3349 (1264; 300)	MD = 83.022	[– 640; 367]	0.671			
LDH—Lactate DeHydrogenase on admission (U/L) Mean ± SD (Median; SE)	746 ± 823 (495; 133)	439 ± 245 (384; 25.9)	MD = 130.243	[– 218; – 46.0]	0.005	1.007	[1.001; 1.011]	0.006
Procalcitonin on admission (ng/mL) Mean ± SD (Median; SE)	4.42 ± 6.14 (2.32; 1.31)	3.50 ± 6.55 (1.41; 0.91)	MD = 1.0432	[– 2.101; 0.222]	0.118			
Lactates on admission (mmol/L) Mean ± SD (Median; SE)	2.51 ± 1.47 (2.15; 0.28)	2.69 ± 1.56 (2.25; 0.16)	MD = 0.1001	[– 0.403; 0.701]	0.598			
Diffuse abdominal pain (N. %)								
No	25 (43.1)	93 (49.2)	OR = 0.782	[0.431; 1.411]	0.416			
Yes	33 (56.9)	96 (50.8)						
Diffuse abdominal rigidity (*N*. %)								
No	44 (75.8)	153 (80.9)	OR = 0.739	[0.362; 1.493]	0.399			
Yes	14 (24.2)	36 (19.1)						
Concomitant choledocholithiasis (*N*. %)								
No	24 (41.4)	130 (68.8)	OR = 1.563	[0.841; 2.851]	0.152			
Yes	34 (58.6)	59 (31.2)						
Concomitant common bile duct obstruction (*N*. %)								
No	52 (89.6)	171 (90.5)	OR = 1.101	[0.412; 2.913]	0.804			
Yes	6 (10.4)	18 (9.5)						
Concomitant acute cholangitis (*N*. %)								
No	43 (74.1)	168 (88.9)	OR = 2.793	[1.332; 5.862]	0.004	3.983	[1.598; 9.930]	0.003
Yes	15 (25.9)	21 (11.1)						
ERCP/ES > 48 h for concomitant choledocholithiasis, common bile duct obstruction, or cholangitis (*N*. %)								
No	19 (67.8)	48 (76.2)	OR = 1.521	[0.561; 4.051]	0.405			
Yes	9 (32.2)	15 (23.8)						
ERCP/ES ≤ 48 h for concomitant choledocholithiasis, common bile duct obstruction, or cholangitis (*N*. %)								
No	17 (44.7)	35 (55.5)	OR = 0.809	[0.324; 2.002]	0.646			
Yes	11 (55.3)	28 (44.5)						
Endoscopic step-up drainage of pancreatic necrosis (*N*. %)								
No	50 (86.2)	141 (74.6)	OR = 0.475	[0.203; 1.061]	0.064	0.339	[0.138; 0.834]	0.018
Yes	8 (13.8)	48 (17.9)						
Upfront open surgical necrosectomy (*N*. %)								
No	37 (63.8)	151 (82.1)	OR = 3.233	[1.723; 6.051]	< 0.001	3.772	[1.912; 7.442]	< 0.001
Yes	21 (36.2)	33 (20.1)						
Percutaneous drainage/minimally invasive necrosectomy (*N*. %)								
No	53 (91.4)	173 (91.5)	OR = 1.021	[0.352; 2.923]	0.970			
Yes	5 (8.6)	16 (8.5)						
Surgical necrosectomy < 2 weeks from the onset (*N*. %)								
No	25 (67.6)	41 (68.3)	OR = 1.043	[0.432; 2.491]	0.937			
Yes	12 (32.4)	19 (31.7)						
Surgical necrosectomy 2–4 weeks from the onset (*N*. %)								
No	21 (59.7)	45 (81.8)	OR = 2.293	[0.954; 5.481]	0.061	1.689	[0.616; 4.633]	0.309
Yes	14 (40.3)	10 (18.2)						
Surgical necrosectomy > 4 weeks from the onset (*N*. %)								
No	28 (77.7)	34 (60.7)	OR = 2.384	[0.953; 5.901]	0.081	0.234	[0.075; 0.724]	0.012
Yes	8 (22.3)	22 (39.3)						
Abdominal compartment syndrome (*N*. %)								
No	43 (74.1)	176 (93.1)	OR = 4.725	[2.092; 10.734]	< 0.001	2.735	[1.090; 6.867]	0.032
Yes	15 (25.9)	13 (6.9)						
Bleeding (*N*. %)								
No	38 (65.5)	165 (87.3)	OR = 3.623	[1.811; 7.221]	< 0.001	2.710	[1.286; 5.712]	0.009
Yes	20 (34.5)	24 (12.7)						
Bowel fistula (*N*. %)								
No	49 (84.5)	177 (93.6)	OR = 2.711	[1.081; 6.803]	0.029	1.085	[0.366; 3.211]	0.884
Yes	9 (15.5)	12 (6.4)						
Necrotizing cholecystitis (*N*. %)								
No	48 (82.7)	181 (95.7)	OR = 4.712	[1.761; 12.634]	0.002	2.669	[0.875; 8.141]	0.084
Yes	10 (17.3)	8 (4.3)						
Antibiotic therapy (*N*. %)								
No	7 (12.1)	28 (14.8)	OR = 1.271	[0.521; 3.073]	0.673			
Yes	51 (87.9)	161 (85.2)						
Antifungal therapy (*N*. %)								
No	33 (56.9)	123 (65.1)	OR = 1.411	[0.771; 2.571]	0.258			
Yes	25 (43.1)	66 (34.9)						
Total Parenteral Nutrition (*N*. %)								
No	33 (56.9)	129 (68.2)	OR = 1.633	[0.893; 2.981]	0.117	0.821	[0.391; 1.722]	0.602
Yes	25 (43.1)	60 (31.8)						
Enteral nutrition (*N*. %)								
No	45 (77.5)	105 (55.5)	OR = 0.361	[0.183; 0.711]	0.003	0.320	[0.143; 0.716]	0.006
Yes	13 (22.5)	84 (44.5)						

*HPB*  Hepato-Pancreato-Biliary, *RAC  *Revised Atlanta Classification, *qSOFA  *quick Sepsis-related Organ Failure Assessment, *BISAP  *Bedside Index of Severity in Acute Pancreatitis, *APACHE II  *Acute Physiology, Age, and Chronic Health Evaluation II, *ERCP/ES*  Endoscopic Retrograde Cholangio-Pancreatography/Endoscopic Sphincterotomy, *WBC  *White Blood Cells

The optimal cut-point was for BMI 34 kg/m^2^ (Sensitivity 13.3%, Specificity 86.96%, PPV 40%, NPV 60.61%, Accuracy 60%), SpO_2_ 91% (Sensitivity 80.39%, Specificity 45.32%, PPV 51.9%, NPV 75.9%, Accuracy 61%), and LDH 554 U/L (Sensitivity 21.43%, Specificity 60.49%, PPV 23.6%, NPV 68.3%, Accuracy 65%).

**Fig. 2:**
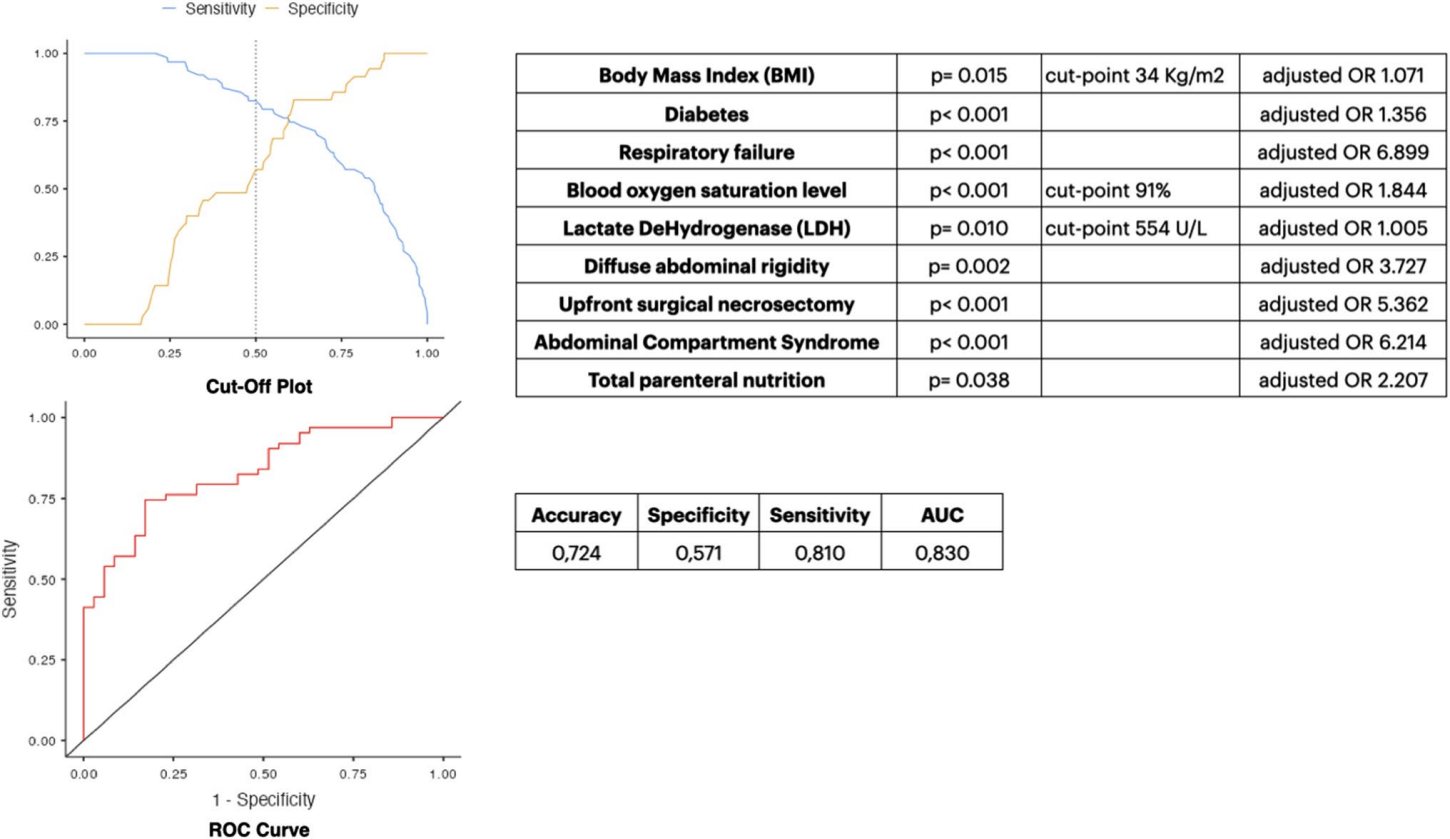
Intensive care unit (ICU) admission prediction model

ROC curves were plotted to assess the performance of the combination of the parameters above to predict ICU admission in patients with IPN. The final stepwise multivariable logistic regression model (logistic regression *X*^2^ 36.3; p < 0.001; pseudo *R*^2^ 0.309; Nagelkerke *R*^2^ 0.425; McFadden’s *R*^2^ 0.284) consisted of 9 variables (Fig. 2). Calibration of the model determined quantitatively by the Hosmer–Lemeshow goodness of fit statistics (LH *X*^2^ 3.07, p = 0.047) confirmed that the model could assign appropriate risk among the patients whose experience is simulated by the model. As a result of discrimination evaluated using ROC analysis, the model’s accuracy was 72.4%, specificity was 57.1%, and sensitivity was 81.0%, with an AUROC = 0.830.

### Predictors of mortality

Overall mortality in the whole cohort of patients with IPN was 23.5%. Factors associated with mortality at univariable analysis are reported in Table 3. Mean age was higher in the non-survivor group than in survivors (*p* = 0.05; MD 5.001). Similarly, the mean CCI (*p* = 0.021; MD 1.003) and BMI (*p* = 0.012; MD 2.701) were higher in the non-survivor group compared to survivors.

Regarding comorbidities, a clinical history of diabetes (*p* = 0.009; OR 1.425) and arterial hypertension (*p* < 0.001; OR 2.954) were more frequent in non-survivors. qSOFA (*p* = 0.002; MD 1.003), BISAP (p = 0.002; MD 1.002), Glasgow-Imrie (*p* = 0.043; MD 1.003) and Ranson’s scores (*p* = 0.064; MD 1.004) were higher in the non-survivors group. The non-survivors group had higher rates of severe AP (*p* < 0.001; OR 3.204), renal failure (*p* < 0.001; OR 3.901), haemodynamic failure (*p* < 0.001; OR 3.864), and respiratory failure (*p* < 0.001; OR 2.823) during hospital admission.

Concerning vital parameters, mean blood oxygen saturation (*p* = 0.019; MD 1.003) was higher in survivors, whereas respiratory rate (*p* = 0.002; MD 2.007) was lower. LDH (*p* = 0.005; MD 130) and CRP levels (p = 0.044; MD 1.401) differed between the survivors and non-survivors groups, with the latter showing higher levels.

Acute cholangitis was more common in non-survivor patients (*p* = 0.004; OR 2.793). ACS (*p* < 0.001; OR 4.725), gastrointestinal and/or intra-abdominal bleeding (*p* < 0.001; OR 3.623), bowel fistula (*p* = 0.029; OR 2.711), necrotising cholecystitis (*p* = 0.002; OR 4.712), and open surgical necrosectomy (*p* < 0.001; OR 3.233) were associated with higher mortality. Being admitted to an HPB surgery department (*p* = 0.044; OR 0.375) and the administration of enteral nutrition (*p* = 0.003; OR 0.361) were protective factors against in-hospital mortality.

Details of the final multivariable prediction model for the risk of mortality in IPN patients are shown in Table 3.

In the multivariable logistic regression analysis, age (*p* = 0.034; aOR 1.030), history of uncontrolled arterial hypertension (*p* = 0.032; aOR 4.245), qSOFA (p = 0.005; aOR 2.828), organ failure (*p* < 0.001; aOR 11.589), renal failure (*p* = 0.022; aOR 2.489), haemodynamic failure (p = 0.018; aOR 2.661), CRP (*p* = 0.044; aOR 1.010), LDH (*p* = 0.006; aOR 1.007), acute cholangitis (*p* = 0.003; aOR 3.983), ACS (p = 0.032; aOR 2.735), gastrointestinal and/or intra-abdominal bleeding (*p* = 0.009; aOR 2.710) and upfront open surgical necrosectomy (*p* < 0.001; aOR 3.772) were identified as independent predictors of mortality. Endoscopic drainage of pancreatic necrosis (*p* = 0.018; aOR 0.339) and delayed (> 4 weeks) necrosectomy (*p* = 0.012; aOR 0.234) were found as protective factors against mortality in the multivariable analysis.

The optimal cut-point was for CRP 125 mg/L (Sensitivity 81.94%, Specificity 38.57%, PPV 80.89%, NPV 40.2%, Accuracy 72.6%), age 76 years (Sensitivity 79.73%, Specificity 39.87%, PPV 82.76%, NPV 30.3%, Accuracy 77.5%), and LDH 510 U/L (Sensitivity 39.21%, Specificity 55.26%, PPV 60.47%, NPV 38.2%, Accuracy 52.3%).

**Fig. 3:**
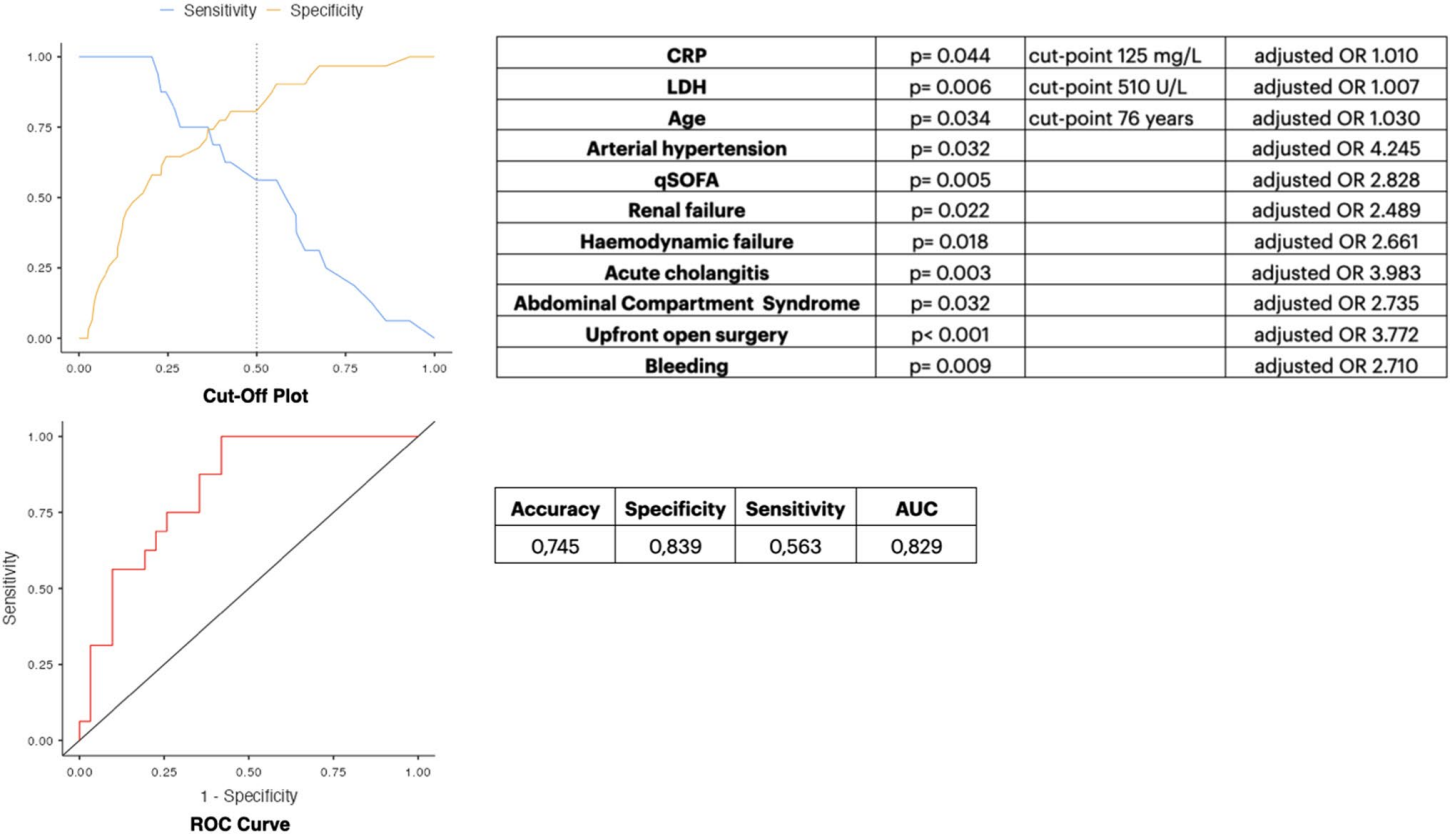
Mortality prediction model

ROC curves were plotted to assess the performance of the combination of the parameters mentioned above to predict mortality in this group of patients. The results of the ROC analysis are shown in Fig. 3. The final results of the stepwise multivariable logistic regression for mortality in patients with IPN (logistic regression *X*^2^ 20.4; *p* = 0.037; pseudo *R*^2^ 0.309; Nagelkerke *R*^2^ 0.423; McFadden’s R^2^ 0.282) consisted of 11 variables. Calibration of the model determined quantitatively by the Hosmer–Lemeshow goodness of fit statistics (LH *X*^2^ 3.04, *p* = 0.067) confirmed that the model could assign appropriate risk among the patients whose experience is simulated by the model. As a result of discrimination evaluated using ROC analysis, the model’s accuracy was 74.5%, specificity was 83.9%, and sensitivity was 56.3%, with an AUROC = 0.829.

## Discussion

In this post hoc analysis of the MANCTRA-1 study, we have shown that the adverse outcomes in patients with IPN are related to two separate groups of factors. On the one hand, we found factors related to the disease course and its severity, such as organ failure, acute cholangitis, and abdominal compartment syndrome, or to the patient’s comorbidity, such as obesity, diabetes, and uncontrolled arterial hypertension. On the other, we found modifiable factors related to patient management. In particular, when the current guidelines and recommendations are not followed, for example, in the cases of upfront open surgical necrosectomy or when nutritional support is provided via total parenteral nutrition instead of enteral nutrition.

While in previous studies [28, 29] predictive variables were assessed to identify early determinants of pancreatic necrosis and organ failure, we implemented our research intending to assess the risk of mortality early in the course of the disease in patients with confirmed IPN.

Although patient-specific risk algorithms have been implemented in previous studies with evidence of benefit in improving the prediction of patient outcomes, it is still undetermined what factors can impact the survival once infection of pancreatic necrosis has been established [30]. In this post hoc analysis of the MANCTRA-1 study, the association of age > 76 years, history of uncontrolled arterial hypertension, CRP > 125 mg/L, LDH > 510 U/L, renal failure, haemodynamic failure and acute cholangitis diagnosed within 72 h from hospital admission allowed to predict mortality. Moreover, by adding the occurrence of necrosectomy performed with open technique, ACS and intra-abdominal bleeding later in the course of the disease, the model could predict mortality with an accuracy of 74.5%. Within this context, the most relevant and potentially modifiable factors to reduce mortality were early haemodynamic and renal support, managing cholangitis with ERCP/ES ≤ 48 h from hospital admission, providing enteral nutrition, and reserving open necrosectomy to patients for whom the minimally invasive and endoscopic step-up approaches have failed to improve clinical conditions. These strategies are currently supported by several clinical guidelines [20, 21].

Previous studies focusing on patients with IPN found that multiple organ failure, long duration (≥ 5 days) of organ failure, and open necrosectomy performed outside a step-up approach were associated with high mortality rates [31]. Similarly, in an extensive systematic review and meta-analysis, Werge et al*.* [8] found that patients with IPN were more than twice as likely to die compared to patients with sterile necrosis. In this setting, the timing of organ failure is essential. In our study, organ failure was detected early in the course of the disease (within 72 h of hospital admission), which can be of absolute relevance in terms of prognosis. In the study by Singh et al*.* [32], among 300 patients with necrotising pancreatitis, 58% had organ failure, in keeping with what we found in our study (62%). The highest mortality was noted in patients with organ failure persisting for longer than three weeks. Moreover, among patients with multiple organ failure, those with multiple sequential failures had a worse outcome than those with simultaneous failures. Unfortunately, in our study, we could not assess if the association of two or more types of organ failure was concomitant or sequential. However, the logistic regression analysis confirmed organ failure as a significant predictor of mortality. Among all types of organ failure, our study showed that renal failure and haemodynamic failure, more than respiratory, were strong predictors of mortality in patients with IPN.

IPN is a disease that mandates individual patient evaluation in a multidisciplinary setting in collaboration among gastroenterologists, surgeons, endoscopists, intensive care physicians, and interventional radiologists, to adequately evaluate patients’ suitability for different available interventions and treatment options. Guidelines recommend that interventional strategies in patients with pancreatic necrosis should be delayed until necrosis is well demarcated [20, 21]. Demarcation facilitates necrosectomy and reduces complications related to drainage and debridement procedures, justifying the recent shift in current practice toward a minimally invasive step-up approach [33, 34]. The PANTER randomised trial by van Santvoort et al*.* [12] showed the advantages of the step-up approach compared to primary open necrosectomy for patients with IPN included lower rates of long-term complications and new-onset organ failure, and less health care resource utilisation. Moreover, in the same study, 35% of patients were successfully treated with percutaneous drainage alone and did not require surgical necrosectomy. It is well established that minimally invasive treatment strategies cause less surgical trauma, including less tissue injury and proinflammatory response in patients who are already severely ill [15]. In clinical practice, this relates to a substantial reduction in the incidence of new-onset multiple organ failure in patients treated with a surgical or endoscopic step-up approach [12, 35, 36]. However, up to 45% of patients treated with a surgical step-up approach develop pancreatico-cutaneous fistulas after percutaneous catheter drainage or minimally invasive necrosectomy as the second step [37, 38]. This is why we are currently witnessing a shift to the endoscopic step-up approach as a treatment preference of IPN whenever possible [39]. In our study, only 22.7% of patients underwent IPN drainage within a step-up endoscopic approach while, contrary to what is recommended by current guidelines, 61.3% underwent upfront open surgical necrosectomy without passing through a step-up strategy. Furthermore, 36.0% of patients who underwent surgical necrosectomy did it in the timing > 4 weeks and 64.0% before four weeks. In our multivariable analysis, while endoscopic drainage of pancreatic necrosis within a step-up approach (aOR 0.339) and delayed (> 4 weeks) necrosectomy (aOR 0.234) were found protective against mortality risk, upfront surgical necrosectomy was associated with four-time increased mortality. Our results were in keeping with previous studies demonstrating that early open surgery is a clear determinant of death risk. At the same time, minimally invasive interventions through a step-up approach, including percutaneous or endoscopic drainage, do not appear to affect mortality [39–41].

Finally, the findings of our study proved that enteral nutrition significantly reduced the risk of ICU admission and the mortality rate. Our results are consistent with some previous data demonstrating the beneficial effect of enteral nutrition over total parenteral nutrition [42]. However, unlike previous studies, we could not assess the effect of enteral nutrition starting at different time points. Patients with severe AP are vulnerable to many potential risk factors associated with the development of pancreatic and/or peri-pancreatic and systemic infections, and receiving total parenteral nutrition has shown to be associated with the risk of developing multi-drug resistant infective complications [43, 44]. Based on these potential advantages, American and European scientific societies of pancreatology currently recommend routine early enteral feeding in all patients with severe AP when patients cannot tolerate an oral diet [21, 45].

### Strengths and limitations

We acknowledge some limitations in this study, mainly related to the retrospective nature of the analysis.

The MANCTRA-1 study included centres having different levels of experience in treating AP. So, it is possible that the risks associated with infections and mortality were more significant if the patients were managed at centres with more limited experience, mainly when critically ill patients were not referred to specialist HPB units. This study was also limited by the variability in practice, different indications for surgical intervention, and quality of the prognostic modelling strategies due to the low adherence level to guidelines recommendations, as demonstrated in our previous audit [24]. These may have introduced the possibility of selection bias.

Nevertheless, the results of our study underlined that the best outcomes in patients with IPN are achieved when the guidelines are followed and that, as for other conditions, the discrepancy between what is recommended and the current daily practice is often significant.

Finally, there is a residual chance of having missed relevant variables, especially those showing a dynamic evolution during the course of the disease. However, there are also several strengths of the present study. First, the strict inclusion criteria of patients with IPN ensured homogeneity in the study population, whereas previous studies looking at different interventions enrolled both infected and non-infected pancreatic collections, which are associated with different mortality rates. Moreover, as a multinational study with 150 participating centres across 41 different countries, the generalisability of our study results is high. Finally, our study emphasised two relevant issues: the need for evidence-based standardisation of the management of IPN and the importance of a timely referral to a specialist unit for patients with extensive necrotizing forms who may require ICU care and 24-h interventional radiological, endoscopic, or HPB surgical services. Indeed, managing patients with IPN involves the availability of many specialty services (gastroenterology, interventional endoscopy, surgery, critical care, and interventional radiology) and the experience of coordinating a multidisciplinary team. Therefore, if the full range of specialists is unavailable in the receiving hospital, a nominated team for managing severe AP patients should coordinate local treatments, where possible, and the referral to a specialist unit where appropriate.

## Conclusions

The results of this post hoc analysis of the MANCTRA-1 study can help overcome current limitations in identifying patients with IPN at the highest risk of death, ultimately leading to early identification of the patients requiring major clinical and interventional efforts. Organ failure (aOR 11.589), acute cholangitis (aOR 3.983), and open surgical necrosectomy (aOR 3.772) were the most significant predictors of mortality. Our study confirmed that, even in a subgroup of particularly ill patients such as those with IPN, upfront open surgery should be avoided as much as possible, as it is a clear determinant of death. Conversely, minimally invasive surgical and endoscopic interventions through a step-up approach should be attempted at the first stage. Patients with IPN should be referred to a specialised centre and taken into a high-dependency or intensive care unit as early as possible.

## Supplementary Information

Below is the link to the electronic supplementary material.

Supplementary file1 (DOC 42 KB)

## Data Availability

The data supporting this study’s findings will be available upon request from the principal investigator [MP].
